# Synthesis and Antitumor Activity Evaluation of Novel Echinatin Derivatives with a 1,3,4-Oxadiazole Moiety

**DOI:** 10.3390/ijms25042254

**Published:** 2024-02-13

**Authors:** Xing Tian, Zihan Sun, Ye Zhong, Huali Yang, Maosheng Cheng, Yang Liu

**Affiliations:** Key Laboratory of Structure-Based Drug Design & Discovery, Ministry of Education, School of Pharmaceutical Engineering, Shenyang Pharmaceutical University, Shenyang 110016, China; 18525738824@163.com (X.T.); kuri_1207@163.com (Z.S.); zy_mc666@sina.com (Y.Z.); yanghl@syphu.edu.cn (H.Y.); mscheng@syphu.edu.cn (M.C.)

**Keywords:** 1,3,4-oxadiazole, echinatin, antiproliferative activity, anti-migration, c-KIT

## Abstract

A series of novel echinatin derivatives with 1,3,4-oxadiazole moieties were designed and synthesized. Most of the newly synthesized compounds exhibited moderate antiproliferative activity against the four cancer cell lines. Notably, Compound **T4** demonstrated the most potent activity, with IC_50_ values ranging from 1.71 µM to 8.60 µM against the four cancer cell lines. Cell colony formation and wound healing assays demonstrated that **T4** significantly inhibited cell proliferation and inhibited migration. We discovered that **T4** exhibited moderate binding affinity with the c-KIT protein through reverse docking. The results were effectively validated through subsequent molecular docking and c-KIT enzyme activity assays. In addition, Western blot analysis revealed that **T4** inhibits the phosphorylation of downstream proteins of c-KIT. The results provide valuable inspiration for exploring novel insights into the design of echinatin-related hybrids as well as their potential application as c-KIT inhibitors to enhance the efficacy of candidates.

## 1. Introduction

The application of natural products in pharmaceutical chemistry has garnered significant attention in recent years. Through the extensive research and development of these compounds, novel perspectives and opportunities can be provided for the design and discovery of innovative drugs [1]. Chalcone is an α,β-unsaturated ketone compound containing two aromatic rings and is a synthetic precursor of flavonoids and isoflavones. Echinatin, a derivative of chalcone, is an active constituent found in licorice; it is obtained from the roots and rhizomes of *Glycyrrhiza* species (*Leguminosae*) and related species [2]. Recent research has substantiated the numerous clinical applications of echinatin, particularly in the realm of inhibiting tumor proliferation [3,4,5,6,7,8,9]. A study conducted by Hong et al. demonstrated that echinatin induced apoptosis and autophagy by inactivating the Akt/mTOR signaling pathway, as revealed through a series of functional assays [10]. In 2021, Lu et al. discovered that echinatin inhibited the proliferation of human tumor cells and induced cell cycle arrest. Moreover, echinatin has been shown to suppress migration and invasion while promoting apoptosis in human tumor cells [11]. A study conducted by Kwak AW et al. in 2019 demonstrated that echinatin induced apoptosis in esophageal squamous cell carcinoma (ESCC) cells [12]. Taken together, the findings of these studies suggest that echinatin may be a novel antitumor agent.

**Figure 1:**
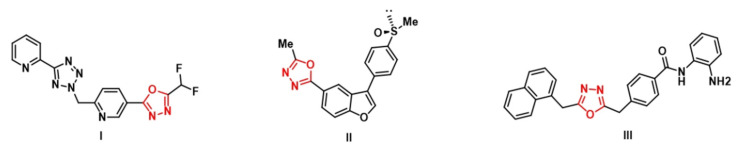
Structural formula of 1,3,4-oxadiazole derivatives with anticancer activity.

The hybrid strategy is a prevalent design approach in the development of novel drug candidates. The advantages of hybrid entities, in comparison to single pharmacophores, include heightened drug activity, improved pharmacokinetic properties, circumvented drug resistance, and diminished toxicity and side effects [13,14]. Over the past few decades, 1,3,4-oxadiazole compounds have garnered significant attention due to their exceptional receptor binding capabilities; they can exist as either mono-substituted or di-substituted forms, and their thermal stability combined with low lipophilicity make them essential anchors in drug design and discovery [15]. Moreover, oxadiazole is frequently employed in drug molecular design as an isosteric substitute for esters and amides [16]. In the molecular docking study conducted by Mohamed et al., it was found that 1,3,4-oxadiazole functioned as a hydrogen bond acceptor, thereby enhancing its interaction with the target protein [15]. A study conducted by Lamya H. et al. in 2021 demonstrated the inhibitory effects of compounds containing a 1,3,4-oxadiazole moiety on the proliferation of human colorectal cancer (HCT116) and human breast cancer (MCF-7) cell lines [17]. A series of compounds reportedly induces G2/M cell cycle arrest and triggers an apoptotic response [18]. As shown in Figure 1, the reported 1,3,4-oxadiazole compounds I, II, and III all displayed excellent inhibitory effects against malignant melanoma, colon cancer, and breast cancer [19,20,21].

**Figure 2:**
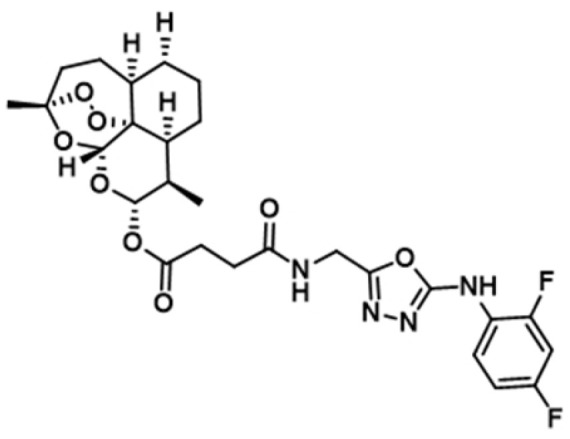
Structure of **A8**.

In our previous study, a series of novel 1,3,4-oxadiazole-artemisinin hybrids were designed and synthesized, and the results of further bioactivity studies demonstrated that most of these compounds exhibited stronger antiproliferative activity than artemisinin [22]. Of these, Compound **A8** (Figure 2) showed the best antitumor activity with an IC_50_ ranging from 4.07 μM to 9.71 μM in four different tumor cell lines. Above all, the significance of the 1,3,4-oxadiazole framework in cancer research has been demonstrated in recent studies, and its derivatives have been reported to exhibit antitumor activity through the inhibition of various enzymes and growth factors [23,24].

**Figure 3:**
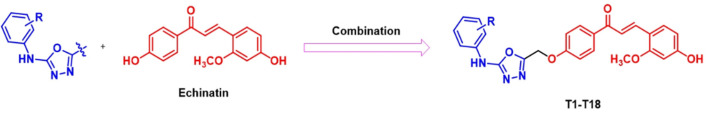
Compounds **T1**–**T18** were designed by the combination strategy.

In this work, to explore whether the introduction of 1,3,4-oxadiazole fragments can enhance the antitumor activity of echinatin, we designed and synthesized a series of echinatin derivatives based on a combination strategy (Figure 3) and evaluated their antitumor activity in vitro. Among these compounds, the consultant Compound **T4** had acceptable antiproliferative effects on the KU812, HT-29, and HCT116 cell lines. Through docking studies and in vitro enzyme activity tests, we found that **T4** inhibited the c-KIT protein and its downstream signaling molecules in a dose-dependent manner. 

## 2. Results and Discussion

### 2.1. Chemistry

**Scheme 1:**
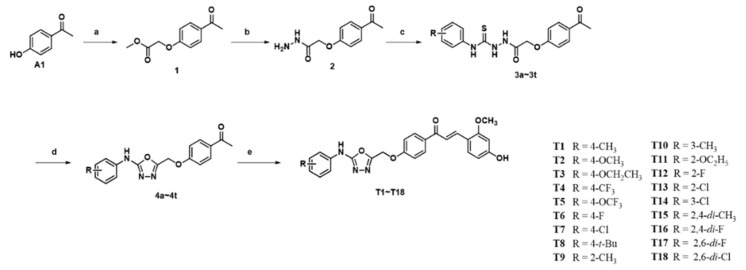
Synthesis of compounds **T1**–**T18**. Reagent and conditions: (a) methyl bromoacetate, K_2_CO_3_, MeCN, 80 °C, 4 h, reflux, 90.1%; (b) 40% hydrazine hydrate, 0 °C, 40 min, 91.2%; (c) aryl isothiocyanate, THF, r.t., 12 h, 43.5%; (d) EDCI·HCl, DMSO, 60 °C, 3 h, 89.4%; (e) 4-hydroxy-2-methoxybenzaldehyde, CuBr_2_, EtOH, 70 °C, 8 h.

Compounds **T1**–**T18** were synthesized from the commercially available **A1** as outlined in Scheme 1. Taking **A1** as the starting material, intermediate 2 was synthesized via substitution and hydrazinolysis. Compound 2 was reacted with different substituted phenyl isothiocyanates to afford target Compounds **3a**–**3r**. By further treatment with EDCI, **3a**–**3r** were converted into intermediates **4a**–**4r**. Finally, target Compounds **T1**–**T18** were successfully obtained via the condensation of **4a**–**4r** with 4-hydroxy-2-methoxybenzaldehyde in EtOH at 70 °C. 

### 2.2. In Vitro Antiproliferative Activity Evaluation

The antiproliferative effects of target Compounds **T1**–**T18** were evaluated in two human colon cancer cell lines (HT-29 and HCT116) and two human hematologic malignant cell lines (K562 and KU812) via a CCK8 assay. Echinatin and adriamycin were co-assayed as controls. The IC_50_ values are presented in Table 1. Notably, Compounds **T1**–**T5**, **T8**, and **T12**–**T14** had antiproliferation effects similar to those of adriamycin and greater than those of echinatin in HT-29 cells, with IC_50_ values of 2.87~13.88 µM. Moreover, **T1**–**T8** displayed activities at the same level as echinatin in KU812 cells, with IC_50_ values of 8.09~17.52 µM, which revealed that the induction of the large site-blocking group at the para position of the benzene could enhance the activity of this series of compounds. When the substituents on the benzene ring were located at the *meta*- and *ortho*-sites, their activity was comparatively inferior to that at the *para*-site, as shown by comparing **T9**–**T14** with **T1**–**T8**. However, any multi-substituted products attached to the R group such as **T15**–**T18** will render the compounds inactive. In particular, Compound **T4** had superior activity against all four tested cancer cell lines, with IC_50_ values of 8.60, 8.09, 2.87, and 1.71 µM. Interestingly, more than half of the compounds exhibited strong inhibitory effects on HCT116 cells, with IC_50_ values ranging from 1.71 to ~10.20 µM, which revealed that HCT116 cells may be more sensitive to these compounds. The most active compound, **T4**, was evaluated for possible cytotoxicity toward the human embryonic intestinal mucosal derived cells line (CCC-HIE-2), and the results (Table 2) indicated that the growth of CCC-HIE-2 cells was not significantly influenced by Compound **T4** (the selectivity index, SI = 16.1). Therefore, **T4** was selected as the most promising compound to explore the preliminary mechanism of HCT116 cell antiproliferation.

### 2.3. Cell Colony Formation and Wound Healing Assays

**Figure 4:**
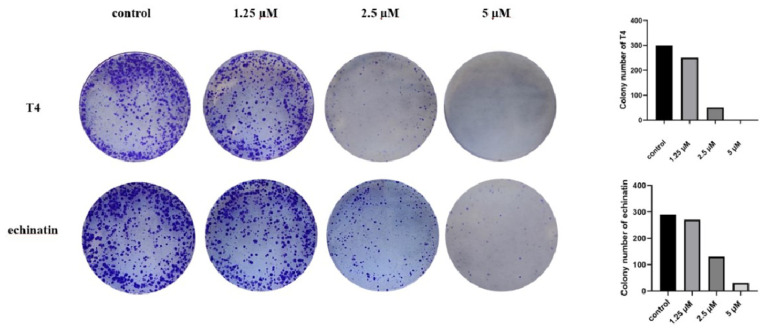
The effect of **T4**, echinatin toward the HCT116 cell colony number; echinatin was used as the control.

**Figure 5:**
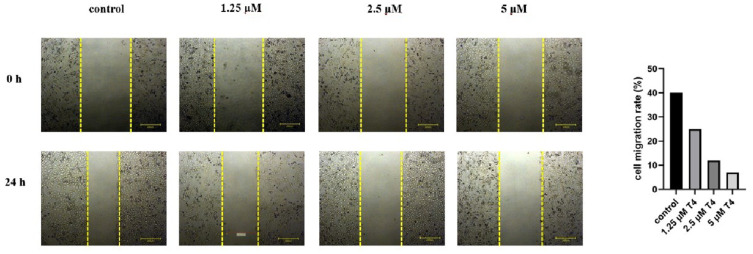
Compound **T4** inhibited the migration of HCT116 cells in a wound healing assay.

The antiproliferation and antimigration activities of Compound **T4** were evaluated through cell colony formation and wound healing assays to more intuitively determine the cellular effects. The cell colony number decreased in a dose-dependent manner, and the antiproliferative effect of **T4** was superior to that of echinatin at the same concentration, as demonstrated in Figure 4. A wound healing assay revealed that **T4** strongly inhibited HCT116 cell migration (Figure 5). Thus, these findings demonstrate that the compound significantly affects the function of HCT116 cells. 

### 2.4. Analysis of Apoptosis by Annexin V-FITC/PI and TUNEL Staining

**Figure 6:**
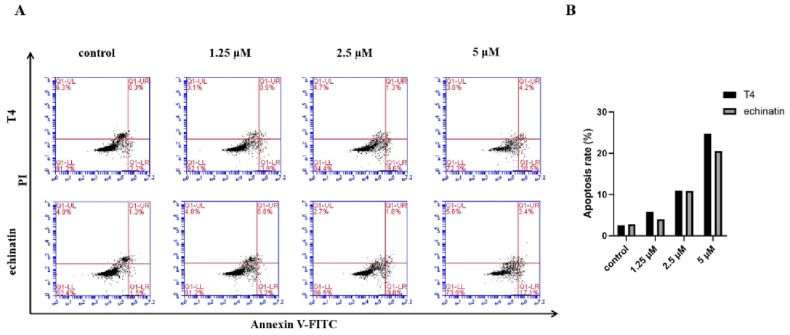
Effect of Compound **T4** and echinatin to induce the apoptosis of HCT116 cells. (**A**) The HCT116 cells were subjected to treatment with the indicated concentrations of compounds for a duration of 48 h. Subsequently, the apoptosis rate was quantified using flow cytometry analysis. (**B**) The distribution of cells within each population was determined as a percentage.

**Figure 7:**
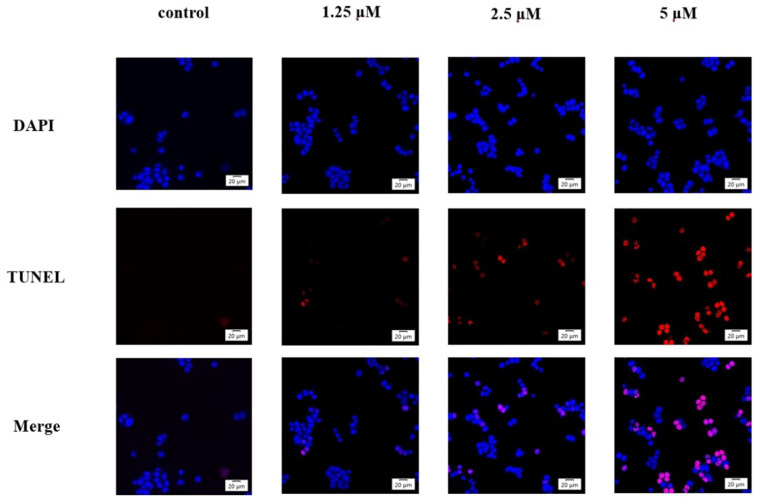
The cell morphological alterations and nuclear changes associated with HCT116 cells were evaluated through TUNEL staining and visualized using fluorescence microscopy following treatment with varying concentrations of **T4**. Bars denote 20 µm.

Compound **T4** was evaluated for its ability to induce apoptosis in HCT116 cells by an Annexin V-FITC/PI binding assay, and it was found that **T4** could increase the apoptosis rate in a concentration-dependent manner. As shown in Figure 6, the apoptosis rate of HCT116 cells increased from 5.8% to 24.7% when HCT116 cells were treated with **T4** at various concentrations, which was slightly greater than that of echinatin (from 4.0% to 20.5%). TUNEL staining revealed a positive correlation between the concentration and the number of cells exhibiting red fluorescence, indicating an increase in apoptotic cells. Conversely, DAPI staining demonstrated a negative correlation between concentration and the number of cells displaying blue fluorescence, suggesting a decrease in normal cells (Figure 7). These results demonstrate that **T4** can cause apoptosis in HCT116 cells.

### 2.5. **T4** Induces ROS Generation and Accumulation

**Figure 8:**
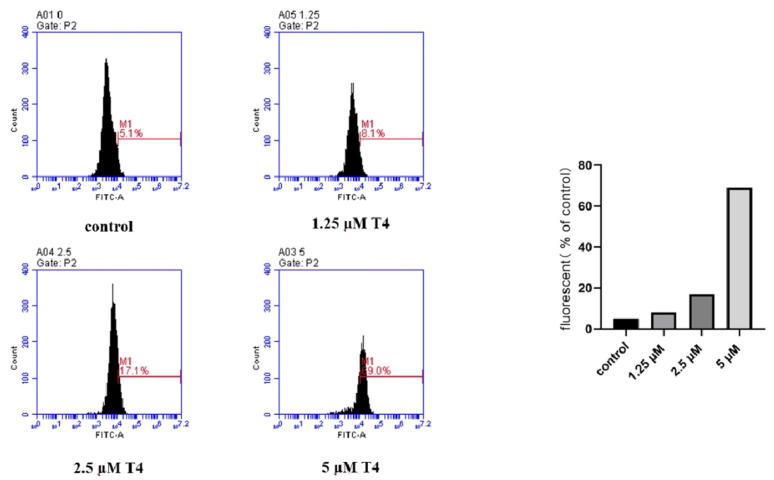
Effects of **T4** on the ROS level of HCT116 cells. HCT116 cells treated with **T4** at the concentrations of 0, 1.25, 2.5, and 5 μM for 48 h and loaded with the DCFH-DA fluorescent probe before analysis.

Mitochondrial dysfunction often accompanies an increase in ROS generation [25,26]. First, we quantified the levels of reactive oxygen species (ROS) using DFCH-DA. As depicted in Figure 8, there was a significant increase in fluorescence intensity with escalating drug concentrations, indicating that **T4** could induce the concentration-dependent generation and accumulation of ROS in HCT116 cells."

### 2.6. Cell Cycle Arrest Induced by Compound T4

**Figure 9:**
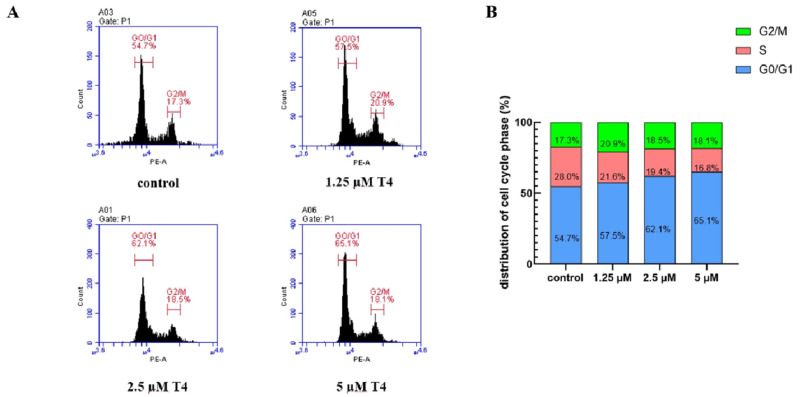
Effect of **T4** on the cell cycle distribution of HCT116 cells. (**A**) HCT116 cells were treated with the indicated concentration of compound **T4** for 48 h. The cell cycle parameters were analyzed by flow cytometry. (**B**) The statistical results of cell cycle distribution.

The antitumor mechanism of Compound **T4** was investigated via cell cycle analysis. The HCT116 cells were treated with the indicated concentrations of **T4** for 48 h and then stained with propidium iodide (PI). The DNA content was measured by flow cytometry. The obtained results were compared with those of nontreated HCT116 cells, which were used as controls. As shown in Figure 9, the treatment of HCT116 cells with **T4** at various concentrations increased the percentage of G0/G1 phase cells from 54.7% to 65.1% and reduced the percentage of S phase cells from 28.0% to 16.8%, suggesting that the antiproliferation activity of **T4** blocked the cell cycle in the G0/G1 phase.

### 2.7. Reverse Docking Analysis

**Figure 10:**
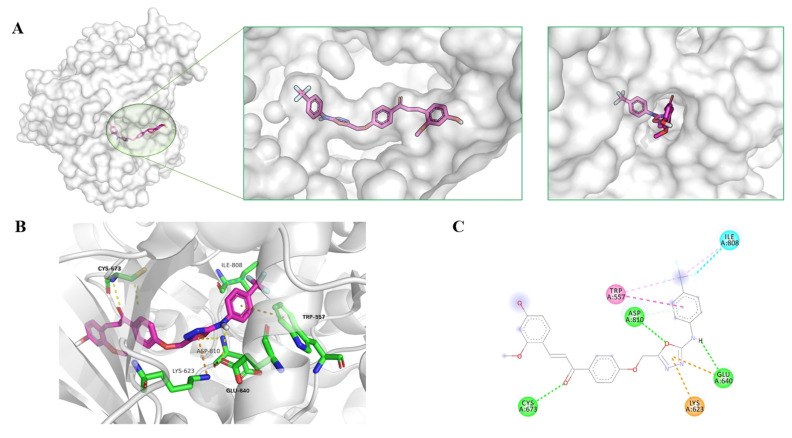
(**A**) The bound conformation of the c-KIT protein (PDB: 4HVS) and **T4**. (**B**) Binding interactions between Compound **T4** and the c-KIT protein (PDB: 4HVS). Yellow dashes represent hydrogen bonds, orange dashes represent π–π interactions, and the blue dash represents halogen bonds. (**C**) Two-dimensional (2D) interactions.

**Figure 11:**
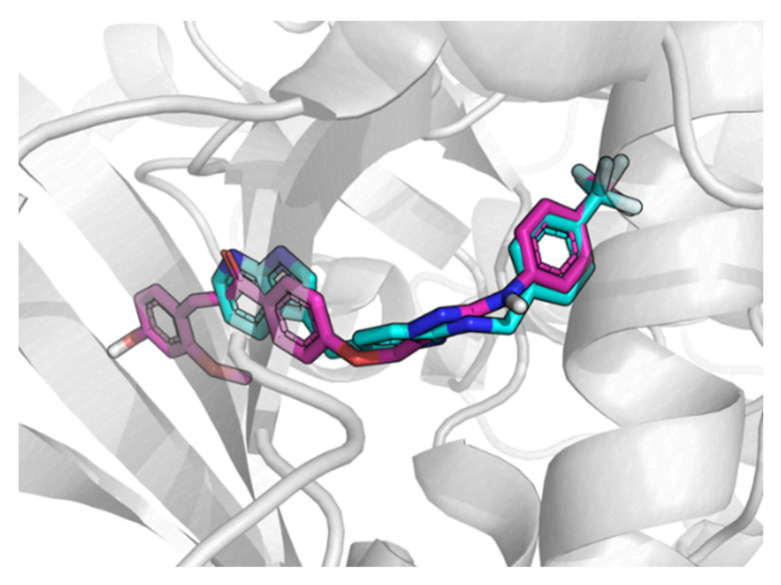
The small molecule inhibitor (PLX647, bule) co-crystallized with the c-KIT protein (PDB: 4HVS) superimposed on Compound **T4** (purple).

Through the aforementioned cell experiments, **T4**, a potent antitumor molecule, was subjected to an exhaustive inverse docking protocol to identify the putative target of this structure. In this technique, a molecule was attempted to dock to the ligand-binding domain of proteins associated with potential activity. According to the results of the current reverse docking protocol, the c-KIT protein (PDB: 4HVS) exhibited the best docking score (binding energy) among the antitumor-related proteins and Compound **T4**, with a docking score of −14.59; refer to the Supplementary Materials for further details (Table S1). The c-KIT proto-oncogene is a well-established oncogenic driver closely implicated in the pathogenesis and progression of human malignancies, rendering it an attractive and promising therapeutic target for novel anticancer interventions. To gain deeper insights into the molecular basis of the inhibitory activity, we further analyzed the docking modes of the tested Compound **T4** with the c-KIT protein (Figure 10A). As shown in Figure 11, Compound **T4** fits well into the c-KIT protein and can be perfectly superimposed onto the small molecule inhibitor (PLX647) of c-KIT. The 1,3,4-oxadiazole ring and amino and carbonyl groups formed three stable hydrogen bonds with Asp138, Glu640, and Cys673 (Figure 11B,C). Additionally, the aryl moiety of **T4** was oriented toward the interior of the binding pocket, establishing π–π interactions with the Trp557 residue of the c-KIT protein. Moreover, the trifluoromethyl group in the benzene ring of **T4** formed a halogen bond with Ile808. These results indicate that **T4** can suppress c-KIT activity.

### 2.8. In Vitro Enzymatic Assay

With the aim of further elucidating the mechanisms responsible for the observed antiproliferative activity of echinatin derivatives, the inhibitory activity of c-KIT was evaluated based on the results of inverse docking studies, as discussed in the previous section. Therefore, we conducted in vitro evaluations of the inhibitory effects of Compounds **T3**, **T4**, and **T8** on c-KIT via the use of PLX647 (a small molecule c-KIT inhibitor [27]) as a positive control. As demonstrated in Table 3, as anticipated, echinatin derivatives had similar activities to those of the positive controls, with IC_50_ values ranging from 0.178 to 0.613 µM. These findings demonstrate that **T4** has a potential inhibitory effect on this target.

### 2.9. Western Blot Analysis and GreenNuc Living Cell Caspase 3 Activity Assay

**Figure 12:**
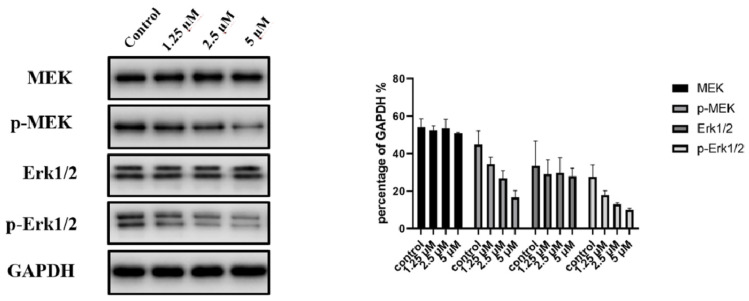
Effects of **T4** on the expression of related proteins of HCT116 cells. Cells were treated with 0, 1.25, 2.5, and 5 µM of Compound **T4** for 48 h, and GAPDH was used as the internal control.

**Figure 13:**
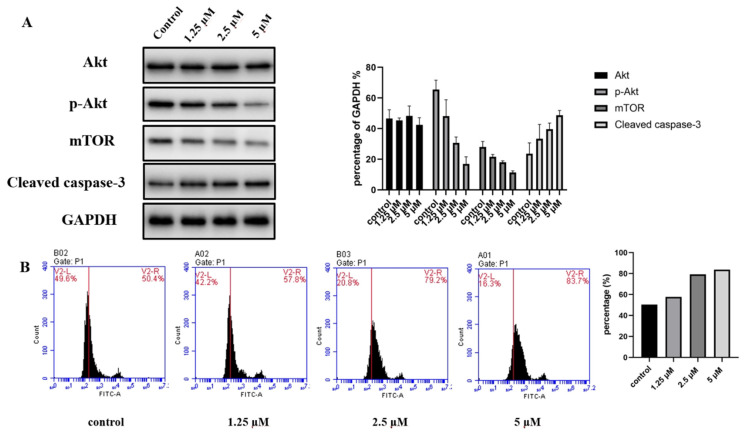
(**A**) The impact of **T4** on the expression of associated proteins in HCT116 cells was investigated. Cells were exposed to varying concentrations (0, 1.25, 2.5, and 5 µM) of Compound **T4** for a duration of 48 h, with GAPDH serving as the internal control. (**B**) Effects of **T4** on living cells in the caspase-3 activity of HCT116 cells.

Based on the results of the reverse docking and enzyme activity tests, we analyzed the expression of c-KIT downstream proteins using a Western blot assay with GAPDH serving as an internal control. Moreover, the downstream effectors of c-KIT-driven malignancies are the MAPK and PI3K pathways [28,29,30]. In this study, HCT116 cells were treated with various concentrations (0, 1.25 μM, 2.5 μM, and 5 μM) of **T4** for 48 h to evaluate its biological effects on downstream targets of c-KIT by Western blot analysis. As shown in Figure 12 and Figure 13, the protein expression levels of Akt, MEK, and Erk1/2 were not significantly altered by treatment with **T4** at the indicated concentrations. In contrast, **T4** significantly downregulated the expression of the phosphorylated forms of Akt, MEK, Erk1/2, and mTOR. In addition, the expression of caspase-3, as a percentage of GAPDH, was significantly upregulated from 24% to 49% following **T4** treatment. Real-time monitoring of caspase-3 activity was performed using the GreenNuc living cell caspase-3 assay kit for live cells from Beyotime Biotechnology. Flow cytometric analysis revealed an observed tendency toward increased caspase 3 activity (Figure 13B). These results demonstrate that Compound **T4** effectively inhibited cell *proliferation by* targeting c-KIT-associated signaling pathways and promoting apoptosis in HCT116 cells.

## 3. Conclusions

In this work, a series of novel echinatin derivatives with 1,3,4-oxadiazole moieties were designed and synthesized. We performed a preliminary screening to assess the anticancer activity of the synthesized compounds. The results of the in vitro antiproliferation assays against four human cancer cell lines demonstrated that the majority of the novel compounds exhibited superior antiproliferative effects compared to those of the control, with a particular sensitivity observed in the HCT116 cell lines. Cell colony and wound healing assays demonstrated that the most promising compound, **T4**, effectively suppressed the proliferation and inhibited the migration of HCT116 cells. Subsequent investigations revealed that **T4** can induce apoptosis and block the cell cycle in a dose-dependent manner. Through reverse docking analysis, it was determined that Compound **T4** exhibited the most favorable binding energy with the c-KIT protein, as evidenced by its significantly low docking energy score of −14.59. The results were effectively validated through subsequent molecular docking and c-KIT enzyme activity assays. In addition, Western blot analysis revealed that **T4** inhibits the phosphorylation of the downstream signaling molecules of c-KIT such as Akt, MEK, and Erk1/2. The results provide valuable inspiration for exploring novel insights into the design of echinatin-related hybrids, as well as their potential application as c-KIT inhibitors to enhance candidate efficacy.

## 4. Methods and Materials

### 4.1. Chemistry

#### 4.1.1. General Information

All reagents (Energy Chemical, Shanghai, China) were used without further purification unless otherwise specified. Solvents were dried and redistilled prior to use in the usual manner. Analytical TLC was performed using silica gel HF254 (Qingdao Haiyang Chemical, Qingdao, Shandong, China). Preparative column chromatography was performed with silica gel H. Melting points were obtained on a Büchi melting point B-540 apparatus (Büchi Labortechnik, Flawil, Switzerland). ^1^H and ^13^C NMR spectra were recorded on a Bruker ARX 600 MHz spectrometer (Bruker, Zurich, Switzerland). Low-resolution electrospray ionization mass spectrometry (ESI-MS) was conducted by an Agilent 6120 (Agilent, Santa Clara, CA, USA). High-resolution ESI-MS was recorded using a Bruker compact mass spectrometer, Agilent G6520 Q-TOF mass spectrometer, or Agilent G6230 TOF LC/MS spectrometer (Agilent, Santa Clara, CA, USA). The NMR spectra are shown in the Supplementary Materials.

#### 4.1.2. General Procedure for the Synthesis of Methyl 2-(4-Acetylphenoxy)Acetate (**1**)

A solution of 4-hydroxyacetophenone (**A1**, 5.0 g, 1.0 eq.) and K_2_CO_3_ (17.0 g, 2.5 eq.) in acetonitrile was stirred at room temperature for 10 min. Methyl bromoacetate (4.2 mL, 43.2 mmol, 1.2 eq.) was added while stirring. The mixture was stirred the refluxing temperature for 4 h until TLC monitoring showed the completion of reactions. The reaction was quenched by the addition of ice-cold water (20 mL) with stirring. The solution was diluted with ethyl acetate and washed with water (3 × 20 mL) and brine (2 × 20 mL). The organic phase was dried over MgSO_4_ and concentrated in vacuum to obtain 6.9 g (yield: 90.0 %) of Compound 1 as a pure white solid; ^1^H NMR (600 MHz, DMSO-d_6_): δ 2.52 (s, 3H), 3.71 (s, 3H), 4.93 (s, 2H), 7.04 (d, *J* = 1.9 Hz, 1H), 7.05 (d, *J* = 1.9 Hz, 1H), 7.92 (d, *J* = 2.0 Hz, 1H), 7.93 (d, *J* = 2.0 Hz, 1H).; ESI-MS (*m*/*z*): 209.1 [M+H]^+^.

#### 4.1.3. General Procedure for the Synthesis of 2-(4-Acetylphenoxy)Acetohydrazide (**2**)

A total of 40% hydrazine hydrate (0.75 mL, 5 eq.) was added to a stirred solution of Compound **1** (0.2 g, 1.0 eq.) in 20 mL methanol. The reaction mixture was stirred at 0 °C for 40 min. After TLC monitoring showed the completion of reactions, the solid product formed was filtered off, washed with water, and dried to furnish 0.18 g (yield: 91.2 %) of Compound **2** as a pure white solid; ^1^H NMR (600 MHz, DMSO-*d*_6_): δ 2.52 (s, 3H), 4.35 (s, 2H), 4.60 (s, 2H), 7.05 (d, *J* = 1.9 Hz, 1H), 7.06 (d, *J* = 1.9 Hz, 1H), 7.92 (d, *J* = 1.9 Hz, 1H), 7.94 (d, *J* = 1.9 Hz, 1H), 9.41 (s, 1H).; ESI-MS (*m*/*z*): 209.1 [M+H]^+^.

#### 4.1.4. General Procedure for the Synthesis of 2-[2-(4-Acetylphenoxy)Acetyl]-N-Arylmethyl-hydrazine-1-carbothioamide (**3a**–**3r**)

A solution of Compound **2** (0.1 g, 1 eq.) and the respective modified phenyl isothiocyanates (1 eq.) was stirred in dry THF at room temperature for 12 h. Then, the solvent was evaporated under reduced pressure and the residue was dispersed in hexane and stirred for 2 h. The solid was filtered in vacuum and dried for use in the next steps without any purification.

#### 4.1.5. General Procedure for the Synthesis of 1-{4-{[5-(arylmethylamino)-1,3,4-oxadiazol-2-yl]methoxy}phenyl}ethan-1-one (**4a**–**4r**)

EDCI (1.2eq) was added to a stirred solution of Compound ****3a**–**3r**** (1 eq.) in 20 mL DMSO. The reaction mixture was stirred at 60 °C for 5 h. Then, the solution was poured into the mixture of ice and water and stirred vigorously for 2 h. The solid were filtered in vacuum and dried for next steps without any purification.

#### 4.1.6. General Procedure for the Synthesis of Compound **T1**–**T18**

A solution of Compound **4a**–**4r** (1 eq.), 2-methoxy-4-hydroxybenzaldehyde (1 eq.) and CuBr_2_ (0.1 eq.) dissolved in EtOH (50 mL) was stirred for 8 h at 70 °C until TLC monitoring showed the completion of reactions. Until the mixture cooled to room temperature, water (20 mL) was added to the reaction mixture and the precipitated material was filtered off. The desired products **T1**–**T18** were purified by flash column chromatography (silica gel).

*(E)-3-(4-Hydroxy-2-methoxyphenyl)-1-{4-{[5-(p-tolylamino)-1,3,4-oxadiazol-2-yl]methoxy} phenyl}propen-one (**T1**)*: Yellow solid; yield: 30.1%; m.p.: 186.1–189.4 °C; ^1^H NMR (600 MHz, DMSO-*d*_6_) δ 10.49 (s, 1H), 10.18 (s, 1H), 8.12 (d, *J* = 8.85 Hz, 2H), 7.97 (d, *J* = 15.57 Hz, 1H), 7.81 (d, *J* = 8.51 Hz, 1H), 7.70 (d, *J* = 15.57Hz, 1H), 7.44 (d, *J* = 8.44 Hz, 2H), 7.22 (d, *J* = 8.87 Hz, 2H), 7.15 (d, *J* = 8.27 Hz, 2H), 6.48–6.46 (m, 2H), 5.44 (s, 2H), 3.85 (s, 3H), 2.25 (s, 3H); ^13^C NMR (150 MHz, DMSO-*d*_6_) δ 187.89, 162.27, 161.29, 161.11, 160.62, 155.94, 139.07, 131.38, 131.03, 130.98, 130.68, 129.94, 118.31, 117.59, 115.24, 115.15, 114.95, 108.70, 99.53, 60.14, 56.02, 20.77; HRMS-ESI (*m*/*z*): calcd. for C_26_H_23_N_3_O_5_, [M+Na]^+^: 480.1535, found 480.1560.

*(E)-3-(4-Hydroxy-2-methoxyphenyl)-1-{4-{{5-[(4-methoxyphenyl)amino]-1,3,4-oxadiazol-2-yl}methoxy}phenyl}p-ropenone (**T2**)*: Yellow solid; yield: 36.1%; m.p.: 212.6–213.6 °C; ^1^H NMR (600 MHz, DMSO-*d*_6_) δ 10.38 (s, 1H), 10.17 (s, 1H), 8.12 (d, *J* = 8.98 Hz, 2H), 7.97 (d, *J* = 15.57 Hz, 1H), 7.81 (d, *J* = 8.52 Hz, 1H), 7.47–7.46 (m, 2H), 7.22 (d, *J* = 8.91 Hz, 2H), 6.93 (d, *J* = 9.05 Hz, 2H), 6.48–6.46 (m, 2H), 5.43 (s, 2H), 3.85 (s, 3H), 3.72 (s, 3H); ^13^C NMR (150 MHz, DMSO-*d*_6_) δ 187.89, 162.27, 161.30, 161.27, 160.61, 155.82, 155.02, 139.06, 132.42, 132.16, 131.02, 130.97, 130.68, 129.13, 119.11, 118.31, 115.24, 114.95, 114.80, 108.70, 99.53, 60.14, 56.02, 55.70; HRMS-ESI (*m*/*z*): calcd. for C_26_H_23_N_3_O_6_, [M+Na]^+^: 496.1485, found 496.1506.

*(E)-1-{4-{{5-[(4-Ethoxyphenyl)amino]-1,3,4-oxadiazol-2-yl}methoxy}phenyl}-3-(4-hydroxy-2-methoxyphenyl) propanone (**T3**)*: Yellow solid; yield: 36.5%; m.p.: 200.1–200.4 °C; ^1^H NMR (600 MHz, DMSO-*d*_6_) δ 10.38 (s, 1H), 10.19 (s, 1H), 8.13 (d, *J* = 8.81 Hz, 2H), 7.98 (d, *J* = 15.64 Hz, 1H), 7.81 (d, *J* = 8.51 Hz, 1H), 7.45 (d, *J* = 8.96 Hz, 2H), 7.22 (d, *J* = 8.83 Hz, 2H), 6.92 (d, *J* = 8.98 Hz, 2H), 6.48–6.46 (m, 2H), 5.43 (s, 2H), 3.86 (s, 3H), 3.98 (q, *J* = 6.95 Hz, 2H), 1.30 (t, *J* = 6.95 Hz, 3H); ^13^C NMR (150 MHz, DMSO-*d*_6_) δ 187.89, 162.27, 161.29, 161.28, 160.62, 155.81, 154.27, 139.07, 132.42, 132.05, 131.03, 130.68, 119.10, 118.29, 115.33, 115.24, 114.95, 108.70, 99.53, 63.63, 60.14, 56.01, 15.16; HRMS-ESI (*m*/*z*): calcd. for C_28_H_27_N_3_O_5_, [M+Na]^+^: 510.1641, found 510.1659. 

(E)-3-(4-Hydroxy-2-methoxyphenyl)-1-{4-{{5-{[4-(trifluoromethyl)phenyl]amino}-1,3,4-oxadiazol-2-yl}methoxy} phenyl}propenone (**T4**): Yellow solid; yield: 15.3%; m.p.: 180.5–181.4 °C; ^1^H NMR (600 MHz, DMSO-d_6_) δ 10.68 (s, 1H), 9.14 (s, 1H), 8.21 (s, 1H), 8.08 (d, *J* = 8.77 Hz, 2H), 8.02 (d, *J* = 15.56 Hz, 1H), 7.97 (d, *J* = 8.75 Hz, 2H), 7.68 (d, *J* = 15.58 Hz, 1H), 7.20 (d, *J* = 8.79 Hz, 2H), 7.07 (d, *J* = 8.77 Hz, 2H), 6.48–6.44 (m, 2H), 5.47 (s, 2H), 3.85 (s, 3H); ^13^C NMR (150 MHz, DMSO-d_6_) δ 196.83, 168.83, 162.24, 161.59, 161.49, 161.04, 160.59, 160.56, 159.39, 156.54, 153.40, 130.98, 126.96, 126.93, 126.14, 119.63, 118.35, 117.49, 115.16, 114.95, 108.69, 108.48, 99.53, 99.22, 65.18, 56.01; HRMS-ESI (*m*/*z*): calcd. for C_26_H_20_F_3_N_3_O_5_, [M+Na]^+^: 534.1253, found 534.1246.

(E)-3-(4-Hydroxy-2-methoxyphenyl)-1-{4-{{5-{[4-(trifluoromethoxy)phenyl]amino}-1,3,4-oxadiazol-2-yl} methoxy}phenyl}propanone (**T5**): Yellow solid; yield: 33.7%; m.p.: 227.6–228.4 °C; ^1^H NMR (600 MHz, DMSO-d_6_) δ 10.87 (s, 1H), 10.18 (s, 1H), 8.13 (d, *J* = 8.83 Hz, 2H), 7.97 (d, *J* = 15.57 Hz, 1H), 7.81 (d, *J* = 8.50 Hz, 1H), 7.70 (d, *J* = 15.58 Hz, 1H), 7.66 (d, *J* = 9.06 Hz, 2H), 7.38 (d, *J* = 8.70 Hz, 2H), 7.23 (d, *J* = 8.85 Hz, 2H), 6.48–6.46 (m, 2H), 5.47 (s, 2H), 3.85 (s, 3H); ^13^C NMR (150 MHz, DMSO-d_6_) δ 196.83, 168.83, 162.24, 161.59, 161.49, 161.04, 160.59, 160.56, 159.39, 156.54, 153.40, 130.98, 126.96, 126.93, 126.14, 119.63, 118.35, 117.49, 115.16, 114.95, 108.69, 108.48, 99.53, 99.22, 65.18, 56.01; HRMS-ESI (*m*/*z*): calcd. for C_26_H_20_F_3_N_3_O_6_, [M+Na]^+^: 550.1202, found 550.1226.

*(E)-1-{4-{{5-[(4-Fluorophenyl)amino]-1,3,4-oxadiazol-2-yl}methoxy}phenyl}-3-(4-hydroxy-2-methoxyphen- yl)propenone (**T6**)*: Yellow solid; yield: 32.6%; m.p.: 211.8–214.1 °C; ^1^H NMR (600 MHz, DMSO-*d*_6_) δ 10.66 (s, 1H), 10.19 (s, 1H), 8.13 (d, *J* = 8.81 Hz, 2H), 7.97 (d, *J* = 15.56 Hz, 1H), 7.81 (d, *J* = 8.50 Hz, 1H), 7.70 (d, *J* = 15.58 Hz, 1H), 7.58–7.57 (m, 2H), 7.21–7.70 (m, 4H), 6.48–6.46 (m, 2H), 5.45 (s, 2H), 3.86 (s, 3H); ^13^C NMR (150 MHz, DMSO-*d*_6_) δ 187.89, 162.28, 161.28, 161.01, 160.62, 156.11, 139.08, 135.38, 132.44, 131.03, 130.68, 119.20, 119.15, 118.29, 116.25, 116.10, 115.24, 114.95, 108.70, 99.53, 60.12, 56.01; HRMS-ESI (*m*/*z*): calcd. for C_25_H_20_FN_3_O_5_, [M+Na]^+^: 484.1285, found 484.1303.

*(E)-1-{4-{{5-[(4-Chlorophenyl)amino]-1,3,4-oxadiazol-2-yl}methoxy}phenyl}-3-(4-hydroxy-2-methoxyphen-yl)propenone (**T7**)*: Yellow solid; yield: 31.2%; m.p.: 211.4–212.1 °C; ^1^H NMR (600 MHz, DMSO-*d*_6_) δ 10.80 (s, 1H), 10.17 (s, 1H), 8.12 (d, *J* = 8.84 Hz, 2H), 7.96 (d, *J* = 15.52 Hz, 1H), 7.80 (d, *J* = 8.49 Hz, 1H), 7.69 (d, *J* = 15.57 Hz, 1H), 7.58 (dd, *J* = 2.65, 9.27 Hz, 2H), 7.41 (d, *J* = 8.83 Hz, 2H), 7.22 (d, *J* = 8.86 Hz, 2H), 6.48–6.44 (m, 2H), 5.46 (s, 2H), 3.85 (s, 3H); ^13^C NMR (150 MHz, DMSO-*d*_6_) δ 187.89, 174.73, 167.43, 162.27, 161.26, 160.76, 160.61, 156.29, 139.07, 137.90, 132.46, 131.15, 131.03, 130.68, 130.11, 129.45, 126.12, 119.12, 118.30, 115.24, 115.16, 114.95, 108.70, 99.53, 60.12, 56.02; HRMS-ESI (*m*/*z*): calcd. for C_25_H_20_ClN_3_O_5_, [M+Na]^+^: 500.0989, found 500.1017.

*(E)-1-{4-{{5-{[4-(tert-butyl)phenyl]amino}-1,3,4-oxadiazol-2-yl}methoxy}phenyl}-3-(4-hydroxy-2-methoxy- phenyl)propanone (**T8**)*: Yellow solid; yield: 35.1%; m.p.: 197.1–198.5 °C; ^1^H NMR (600 MHz, DMSO-*d*_6_) δ 10.50 (s, 1H), 10.17 (s, 1H), 8.13 (d, *J* = 8.87 Hz, 2H), 7.97 (d, *J* = 15.57 Hz, 1H), 7.81 (d, *J* = 8.52 Hz, 1H), 7.46 (d, *J* = 8.75 Hz, 2H), 7.36 (d, *J* = 8.75 Hz, 2H), 7.23 (d, *J* = 8.88 Hz, 2H), 6.48–6.46 (m, 2H), 5.44 (s, 2H), 3.85 (s, 3H), 1.26 (s, 9H); ^13^C NMR (150 MHz, DMSO-*d*_6_) δ 187.89, 162.27, 161.30, 161.15, 160.62, 155.95, 144.87, 139.07, 136.36, 132.43, 131.03, 130.68, 126.19, 118.31, 117.39, 115.25, 114.96, 108.70, 99.53, 60.14, 56.02, 34.39, 31.69; HRMS-ESI (*m*/*z*): calcd. for C_29_H_29_N_3_O_5_, [M+Na]^+^: 522.2005, found 522.2025.

*(E)-3-(4-Hydroxy-2-methoxyphenyl)-1-{4-{[5-(o-tolylamino)-1,3,4-oxadiazol-2-yl]methoxy} phenyl}propanone (**T9**)*: Yellow solid; yield: 29.2%; m.p.: 206.5–209.2 °C; ^1^H NMR (600 MHz, DMSO-*d*_6_) δ 10.19 (s, 1H), 9.65 (s, 1H), 8.12 (d, *J* = 8.77 Hz, 2H), 7.97 (d, *J* = 15.56 Hz, 1H), 7.81 (d, *J* = 8.50 Hz, 1H), 7.71–7.68 (m, 2H), 7.22–7.21 (m, 4H), 7.05–7.03 (m, 1H), 6.48–6.46 (m, 2H), 5.42 (s, 2H), 3.85 (s, 3H), 2.27 (s, 3H); ^13^C NMR (150 MHz, DMSO-*d*_6_) δ 187.90, 162.31, 162.25, 161.28, 160.62, 156.24, 139.08, 136.94, 132.43, 131.11, 131.02, 130.69, 129.61, 126.97, 124.46, 121.56, 118.29, 115.21, 114.94, 108.72, 99.54, 60.19, 56.02, 18.31; HRMS-ESI (*m*/*z*): calcd. for C_26_H_23_N_3_O_5_, [M+Na]^+^: 480.1535, found 480.1553.

*(E)-3-(4-Hydroxy-2-methoxyphenyl)-1-{4-{[5-(m-tolylamino)-1,3,4-oxadiazol-2-yl]methoxy} phenyl}propen-one (**T10**)*: Yellow solid; yield: 26.6%; m.p.: 187.6–190.8 °C; ^1^H NMR (600 MHz, DMSO-*d*_6_) δ 10.53 (s, 1H), 10.18 (s, 1H), 8.12 (d, *J* = 8.76 Hz, 2H), 7.97 (d, *J* = 15.57 Hz, 1H), 7.81 (d, *J* = 8.41 Hz, 1H), 7.70 (d, *J* = 15.58 Hz, 1H), 7.35 (d, **J* =* 8.06 Hz, 2H), 7.22–7.21 (m, 3H), 6.83 (d, *J* = 7.46 Hz, 1H), 6.48–6.46 (m, 2H), 5.45 (s, 2H), 3.85 (s, 3H), 2.30 (s, 3H); ^13^C NMR (150 MHz, DMSO-*d*_6_) δ 187.89, 162.27, 161.28, 161.02, 160.62, 156.03, 139.07, 138.85, 132.44, 131.03, 130.69, 129.41, 123.26, 118.31, 117.99, 115.24, 114.95, 114.78, 108.70, 99.53, 60.15, 56.02, 21.72; HRMS-ESI (*m*/*z*): calcd. for C_26_H_23_N_3_O_5_, [M+Na]^+^: 480.1535, found 480.1558.

*(E)-1-{4-{{5-[(2-Ethoxyphenyl)amino]-1,3,4-oxadiazol-2-yl}methoxy}phenyl}-3-(4-hydroxy-2-methoxyphe-nyl)propenone (**T11**)*: Yellow solid; yield: 33.4%; m.p.: 175.4–188.3 °C; ^1^H NMR (600 MHz, DMSO-*d*_6_) δ 10.18 (s, 1H), 9.64 (s, 1H), 8.12 (d, *J* = 8.29 Hz, 2H), 7.97 (d, *J* = 15.57 Hz, 1H), 7.91 (d, *J* = 7.92 Hz, 1H), 7.81 (d, *J* = 8.45 Hz, 1H), 7.70 (d, *J* = 15.59 Hz, 1H), 7.22 (d, *J* = 8.29 Hz, 2H), 7.03 (d, *J* = 4.26 Hz, 2H), 6.97–6.94 (m, 2H), 6.48–6.46 (m, 2H), 5.43 (s, 2H), 4.67 (q, *J* = 6.87 Hz, 6.87 Hz, 6.88 Hz, 2H), 3.86 (s, 3H), 1.35 (t, *J* = 6.89 Hz, 6.89 Hz, 3H); ^13^C NMR (150 MHz, DMSO-*d*_6_) δ 187.90, 162.27, 161.83, 161.26, 160.62, 156.21, 148.79, 139.07, 132.42, 131.02, 130.69, 127.79, 124.03, 120.90, 119.88, 118.32, 115.20, 114.96, 112.67, 108.71, 99.53, 64.37, 60.15, 56.02, 14.98; HRMS-ESI (*m*/*z*): calcd. for C_27_H_25_N_3_O_6_, [M+Na]^+^: 510.1641, found 510.1672.

*(E)-1-{4-{{5-[(2-Fluorophenyl)amino]-1,3,4-oxadiazol-2-yl}methoxy}phenyl}-3-(4-hydroxy-2-methoxyphen-yl)propenone (**T12**)*: Yellow solid; yield: 23.7%; m.p.: 186.2–187.4 °C; ^1^H NMR (600 MHz, DMSO-*d*_6_) δ 10.44 (s, 1H), 10.17 (s, 1H), 8.13 (d, *J* = 8.89 Hz, 2H), 8.13–8.11 (m, 1H), 7.97 (d, *J* = 15.53 Hz, 1H), 7.80 (d, **J* =* 8.52 Hz, 1H), 7.69 (d, *J* = 15.59 Hz, 1H), 7.40–7.37 (m, 1H), 7.22 (dd, *J* = 7.70, 5.76 Hz, 3H), 6.48–6.44 (m, 2H) 5.44 (s, 2H), 3.85 (s, 3H); ^13^C NMR (150 MHz, DMSO-*d*_6_) δ 187.91, 174.99, 167.45, 162.29, 161.35, 160.63, 156.65, 152.11, 139.08, 132.44, 131.03, 130.12, 129.14, 125.22, 121.40, 118.30, 116.12, 115.99, 115.24, 114.95, 108.70, 65.50, 56.02; HRMS-ESI (*m*/*z*): calcd. for C_25_H_20_FN_3_O_5_, [M+Na]^+^: 484.1285, found 484.1308.

*(E)-1-{4-{{5-[(2-Chlorophenyl)amino]-1,3,4-oxadiazol-2-yl}methoxy}phenyl}-3-(4-hydroxy-2-methoxyphen-yl)propenone (**T13**)*: Yellow solid; yield: 37.9%; m.p.: 191.3–191.8 °C; ^1^H NMR (600 MHz, DMSO-*d*_6_) δ 10.18 (s, 1H), 10.06 (s, 1H), 8.13 (d, *J* = 8.88 Hz, 2H), 7.9 (m, 2H), 7.81 (d, *J* = 8.51 Hz, 1H), 7.70 (d, *J* = 15.58 Hz, 1H), 7.51 (dd, *J* = 1.21, 7.99 Hz, 1H), 7.40–7.37 (m, 1H), 7.22 (d, *J* = 8.89 Hz, 2H), 7.17–7.15 (m, 1H), 6.49–6.45 (m, 2H), 5.44 (s, 2H), 3.86 (s, 3H); ^13^C NMR (150 MHz, DMSO-*d*_6_) δ 187.89, 162.28, 161.73, 161.25, 160.62, 156.80, 139.08, 135.54, 132.44, 131.03, 130.69, 130.30, 128.39, 125.61, 124.77, 123.03, 118.30, 115.23, 114.95, 108.70, 99.53, 60.13, 56.02; HRMS-ESI (*m*/*z*): calcd. for C_25_H_20_ClN_3_O_5_, [M+Na]^+^: 500.0989, found 500.1003.

*(E)-1-{4-{{5-[(3-Chlorophenyl)amino]-1,3,4-oxadiazol-2-yl}methoxy}phenyl}-3-(4-hydroxy-2-methoxyphen-yl)propenone (**T14**)*: Yellow solid; yield: 22.4%; m.p.: 225.0–225.9 °C; ^1^H NMR (600 MHz, DMSO-*d*_6_) δ 10.90 (s, 1H), 10.18 (s, 1H), 8.13 (d, *J* = 8.83 Hz, 2H), 7.97 (d, *J* = 15.55 Hz, 1H), 7.81 (d, *J* = 8.51 Hz, 1H), 7.73–7.71 (m, 1H), 7.70 (d, *J* = 15.59 Hz, 1H), 7.45 (dd, *J* = 1.31, 8.20 Hz, 1H), 7.38–7.37 (m, 1H), 7.23 (d, *J* = 8.86 Hz, 2H), 7.09–7.06 (m, 1H), 6.48–6.46 (m, 2H), 5.47 (s, 2H), 3.85 (s, 3H); ^13^C NMR (150 MHz, DMSO-*d*_6_) δ 187.89, 162.28, 161.25, 160.62, 156.41, 140.37, 139.08, 134.00, 132.46, 131.27, 131.04, 130.69, 122.17, 118.29, 116.95, 116.16, 115.23, 114.95, 108.70, 99.53, 60.11, 56.02; HRMS-ESI (*m*/*z*): calcd. for C_25_H_20_ClN_3_O_5_, [M+Na]^+^: 500.0989, found 500.1005.

*(E)-1-{4-{{5-[(2,4-Dimethylphenyl)amino]-1,3,4-oxadiazol-2-yl}methoxy}phenyl}-3-(4-hydroxy-2-methoxy-phenyl)propenone (**T15**)*: Yellow solid; yield: 32.7%; m.p.: 192.9–194.1 °C; ^1^H NMR (600 MHz, DMSO-*d*_6_) δ 10.17 (s, 1H), 9.54 (s, 1H), 8.12 (d, *J* = 8.79 Hz, 2H), 7.97 (d, *J* = 15.57 Hz, 1H), 7.81 (d, *J* = 8.50 Hz, 1H), 7.70 (d, *J* = 15.58 Hz, 1H), 7.52 (d, *J* = 8.09 Hz, 1H), 7.21 (d, *J* = 8.81 Hz, 2H), 7.03–7.01 (m, 2H), 6.48–6.46 (m, 2H), 5.40 (s, 2H), 3.85 (s, 3H), 2.24 (s, 3H), 2.22 (s, 3H); ^13^C NMR (150 MHz, DMSO-*d*_6_) δ 187.89, 162.48, 162.27, 161.29, 160.62, 156.10, 139.07, 134.35, 133.72, 132.41, 131.65, 131.02, 130.68, 129.96, 127.37, 122.12, 118.31, 115.21, 114.96, 108.70, 99.53, 60.18, 56.02, 20.81, 18.20; HRMS-ESI (*m*/*z*): calcd. for C_27_H_25_N_3_O_5_, [M+Na]^+^: 494.1692, found 494.1708.

*(E)-1-{4-{{5-[(2,4-Difluorophenyl)amino]-1,3,4-oxadiazol-2-yl}methoxy}phenyl}-3-(4-hydroxy-2-methoxy-phenyl)propenone (**T16**)*: Yellow solid; yield: 36.7%; m.p.: 223.4–223.9 °C; ^1^H NMR (600 MHz, DMSO-*d*_6_) δ 10.42 (s, 1H), 10.18 (s, 1H), 8.12 (d, *J* = 8.86 Hz, 2H), 7.97 (d, *J* = 15.58 Hz, 1H), 7.81 (d, *J* = 8.51 Hz, 1H), 7.70 (d, **J* =* 15.57 Hz, 1H), 7.38–7.36 (m, 1H), 7.22 (d, *J* = 8.87 Hz, 2H), 7.15–7.13 (m, 2H), 6.48–6.46 (m, 2H), 5.44 (s, 2H), 3.86 (s, 3H); ^13^C NMR (150 MHz, DMSO-*d*_6_) δ 187.89, 162.28, 161.52, 161.24, 160.62, 156.67, 139.08, 132.44, 131.02, 130.68, 122.90, 122.84, 118.29, 115.23, 114.95, 111.93, 111.91, 111.78, 111.76, 108.70, 105.03, 104.87, 104.85, 104.70, 99.53, 60.10, 56.01; HRMS-ESI (*m*/*z*): calcd. for C_25_H_19_F_2_N_3_O_5_, [M+Na]^+^: 502.1190, found 502.1222.

*(E)-1-{4-{{5-[(2,6-Difluorophenyl)amino]-1,3,4-oxadiazol-2-yl}methoxy}phenyl}-3-(4-hydroxy-2-methoxy-phenyl)propenone (**T17**)*: Yellow solid; yield: 37.1%; m.p.: 193.0–194.3 °C; ^1^H NMR (600 MHz, DMSO-*d*_6_) δ 10.17 (s, 2H), 8.11 (d, *J* = 8.91Hz, 2H), 7.97 (d, *J* = 15.58 Hz, 1H), 7.81 (d, *J* = 8.53 Hz, 1H), 7.70 (d, *J* = 15.57 Hz, 1H), 7.40–7.37 (m, 1H), 7.22–7.21 (m, 4H), 6.49–6.46 (m, 2H), 5.40 (s, 2H), 3.85 (s, 3H); ^13^C NMR (150 MHz, DMSO-*d*_6_) δ 187.89, 162.28, 161.73, 161.25, 160.62, 156.80, 139.08, 135.54, 132.44, 131.03, 130.69, 130.30, 128.39, 125.61, 124.77, 123.03, 118.30, 115.23, 114.95, 108.70, 99.53, 60.13, 56.02; HRMS-ESI (*m*/*z*): calcd. for C_25_H_19_F_2_N_3_O_5_, [M+Na]^+^: 502.1190 found, 502.1222.

*(E)-1-{4-{{5-[(2,6-Dichlorophenyl)amino]-1,3,4-oxadiazol-2-yl}methoxy}phenyl}-3-(4-hydroxy-2-methoxy-phenyl)propenone (**T18**)*: Yellow solid; yield: 35.1%; m.p.: 172.3–174.6 °C; ^1^H NMR (600 MHz, DMSO-*d*_6_) δ 10.26 (s, 1H), 10.18 (s, 1H), 8.11 (d, *J* = 8.88 Hz, 2H), 7.97 (d, *J* = 15.57 Hz, 1H), 7.81 (d, *J* = 8.52 Hz, 1H), 7.70 (d, *J* = 15.58 Hz, 1H), 7.59 (d, *J* = 7.60 Hz, 2H), 7.38 (s, 1H), 7.20 (d, *J* = 8.88 Hz, 2H), 6.48–6.46 (m, 2H), 5.39 (s, 2H), 3.85 (s, 3H); ^13^C NMR (150 MHz, DMSO-*d*_6_) δ 187.89, 170.80, 162.26, 161.24, 160.61, 139.05, 132.42, 130.99, 130.68, 129.50, 118.31, 115.24, 114.96, 108.70, 99.53, 60.22, 56.02; HRMS-ESI (*m*/*z*): calcd. for C_25_H_19_Cl_2_N_3_O_5_, [M+Na]^+^: 534.0599, found 534.0624.

### 4.2. Pharmacology

#### 4.2.1. Cell Cytotoxicity Assay

For all biological assays, all of the synthesized compounds were dissolved in DMSO. The cytotoxicity of all of the target compounds against the HCT116, K562, KU812, and HT-29 cells was determined by an CCK8 assay in vitro using adriamycin and echinatin as the controls. About 3000 cells per well were seeded in a 96-well plate and incubated for 24 h. Then, the cells were stimulated with different concentrations of test compounds for 48 h. The CCK8 solution (100 µL 0.5 mg/mL^−1^) was added to each well, and the cells were incubated for another 4 h. Then, the absorbance of the samples was measured at 450 nm. The IC_50_ values were calculated according to the Logit method after obtaining the inhibitory rate. All experiments were repeated three times.

#### 4.2.2. Cell Colony Formation Assay

To investigate the effects of echinatin derivatives on the clonogenic potential of HCT116 cells, the colony formation assay was performed as previously described. HCT116 cells were seeded into 6-well plates at 37 °C with 5% CO_2_ for 24 h and then treated with Compound **T4** and echinatin at various concentrations (0 µM, 1.25 µM, 2.5 µM, and 5 µM) for 14 d. Then, cells were stained with crystal violet solution and clone numbers were counted directly with the naked eye [31].

#### 4.2.3. Wound Healing Assay

Five lines were drawn evenly on the back of the 6-well plate and transferred HCT116 cells (1 × 10^6^/well) in a 6-well plate until a uniform monolayer of cells formed at the bottom of the 6-well plate. Then, we drew lines on the cells using a 200 μL pipette tip. We then carefully washed the damaged cell monolayer with PBS, then treated with different concentrations of Compound **T4**, and incubated in serum-free medium for 24 h. We then used an inverted microscope to take photos of the 6-hole plate at the 0 and 24 h time points and used ImageJ for processing and analysis [22].

#### 4.2.4. Cell Apoptosis Assay

Approximately 4 × 10^5^ cells per well were seeded in a 6-well plate and incubated for 12 h, then the discarded medium was replaced with fresh medium containing different concentrations of Compound **T4** (0 μM, 1.25 μM, 2.5 μM, and 5 μM) for another 48 h. After incubation, the cells were harvested and washed twice with cold PBS. The obtained cells were stained using a Annexin V-FITC/PI Apoptosis Detection Kit (Beyotime, Haimen, China) according to the manufacturer’s instructions. After that, the samples were analyzed by flow cytometry (Beckman Coulter cytoFLEX, Brea, CA, USA).

#### 4.2.5. TUNEL Staining

The slides were immersed in 4% paraformaldehyde (pH 7.4) for 25 min at room temperature and then washed with PBS 3 times. The cells were immersed in 0.1% Triton X-100 solution prepared with PBS for 10 min (operation on ice) and then washed twice with PBS. Dilute 5× equilibration buffer with deionized water and 100 µL 1× equilibration buffer were added to each climbing tablet to cover the sample area to be tested, then incubated at room temperature for 15 min. After the buffer solution of 1 µD was added to the buffer solution, most of the buffer solution was added to the buffer solution of 1 µD, then the buffer solution was used to wash off the buffer. The slides were placed in a wet box and incubated at 37 °C for 60 min. The wet box was wrapped with aluminum foil to avoid light. These were then washed three times with PBS with DAPI dripped and incubated in the dark for 5 min. The nucleus of the specimen was stained. Water-absorbent paper was used to absorb the liquid on the climbing sheet, then a sealing liquid containing an anti-fluorescence quenching agent was used to seal the film before the images were observed and collected under a fluorescence microscope [22].

#### 4.2.6. Cell Cycle Assays

The effects of Compound **T4** on cell cycle progression were verified by using a standard (PI) staining procedure followed by flow cytometry analysis. HCT116 cells were seeded in 6-well plates and incubated for 24 h until the cells adhered. The cells were treated with various concentrations of Compound F8 and cultured for 72 h. Control cells and treated cells were washed twice with PBS, centrifuged (1000 rpm, 5 min), and collected at a final concentration of 50,000 cells. Then, a single cell suspension was fixed in ice-cold 70% (*v*/*v*) ethanol overnight at 4 °C. The cells were washed again with PBS. A total of 100 μL RNase A was added and the cells were incubated at 37 °C for 30 min. The cell DNA was stained with 400 μL PI for 30 min. Data collection and analysis were carried out using a flow cytometer (Beckman Coulter cytoFLEX, USA) [32].

#### 4.2.7. Intracellular ROS Generation

Approximately 4 × 10^5^ cells per well were seeded in a 6-well plate and incubated for 12 h, then the discarded medium was replaced with fresh medium containing different concentrations of Compound **T4** (0 μM, 0.625 μM, 1.25 μM, 2.5 μM, and 5 μM) for 48 h. ROS production was detected by using the peroxide-sensitive fluorescent probe DCFH-DA. After treatment with different concentrations of **T4**, the cells were incubated with 10 µg/mL DCFH-DA at 37 °C for 20 min. Then, the cells were harvested. Samples were analyzed by flow cytometry (FACS Calibur, Becton-Dickinson, East Rutherford, NJ, USA).

#### 4.2.8. Docking Study

Molecular docking studies were performed to evaluate the prospective interaction between the compound and the target receptors. The inhibition profiles of the compounds against c-KIT were investigated by molecular docking. In silico studies were performed using the Maestro 13.5 program of the Schrödinger Molecular Modeling Suite. Initially, the X-ray crystal structures of target proteins were obtained from RCSB PDB: c-KIT (PDB ID: 4HVS). Compounds were drawn using Chem-draw and transferred to Schrödinger for optimization studies using Maestro’s LigPrep software (v9.9) at physiological pH and we prepared possible stereoisomers and salts. The graphical representations in all figures were rendered using Pymol v2.5.

#### 4.2.9. Biochemical c-KIT Activity Assay

The enzymatic activities of c-KIT were determined by in vitro kinase assays using the ADP-Glo reagent (Promega, Madison, WI, USA) according to the manufacturer’s instructions. Briefly, 250 ng of c-KIT protein was incubated with compounds in 10 μL of reaction buffer (40 mM Tris−HCl, pH 7.4, 2 mM MnCl_2_, 2 mM DTT, 20 mM MgCl_2_, 0.1 mg/mL bovine serum albumin, 1 mM Na_3_VO_4_) containing 20 μΜ ATP and 40 μM poly (Glu_4_-Tyr_1_) as substrates at 30 °C for 150 min. The ADP-Glo reagent was then added, and the mixture was incubated at 25 °C for 40 min. The kinase detection reagent was added for another 30 min at 25 °C. The converted ADP was determined by luminescence measurement using a Wallac Vector 1420 multilabel counter (PerkinElmer).

#### 4.2.10. Western Blotting Assay

HCT116 cells were seeded at a density of 4 × 10^5^ cells per well and treated with various concentrations of Compound **T4** (0, 1.25, 2.5, and 5 µM) for 48 h. After this, cells were collected and washed twice with ice-cold DPBS. The pellets were resuspended in a total protein extraction buffer (20 mM HEPES, 350 mM NaCl, 20% glycerol, 1% NP-40, 1 mM MgCl_2_, 0.5 mM EDTA, 0.1 mM EGTA, 0.1 mM DTT, 0.1 mM PMSF) containing a protease inhibitor cocktail and incubated on ice for 30 min with intermittent mixing. The protein concentration was measured using the Bradford reagent (Bio-Rad Laboratories Inc., Hercules, CA, USA). An equal amount (20 µg) of protein was loaded on 10% poly acrylamide gels and transferred to a nitrocellulose membrane. After blocking with 5% skimmed milk, the membrane was incubated at 4 °C overnight with specific primary antibodies. The membrane was washed and incubated at room temperature for 1 h with secondary antibodies conjugated with horseradish peroxidase (HRP). Finally, the immunoblot was developed for visualization using a chemiluminescence kit. Primary antibodies for Cleaved-caspase 3, MEK, p-MEK, Erk1/2, p-Erk1/2, mTOR, Akt, p-Akt, and GAPDH and all secondary antibodies were obtained from Santa Cruz Biotechnology (Santa Cruz, CA, USA) [22].

#### 4.2.11. GreenNuc Living Cell Caspase 3 Activity Assay

HCT116 cells were seeded at a density of 4 × 10^5^ cells per well and treated with various concentrations of Compound **T4** (0, 1.25, 2.5, and 5 µM) for 48 h. Subsequently, the cells were incubated with GreenNuc™ Caspase-3 Substrate and finally analyzed with bNMy flow cytometry (Beckman Coulter cytoFLEX, USA).

#### 4.2.12. Statistical Analysis 

Values are presented as the means ± SD from at least three independent experiments. Statistical analyses were performed using GraphPad Prism 8. The flow cytometry-related experiment was analyzed by C Flow Plus. The cell colony formation assay and Western blotting were measured by Image J (x64) 2019.

## Figures and Tables

**Table 1:** IC_50_^a^ values (μM) of Compounds TI–T18, echinatin, and adriamycin against four human cancer cell lines.

Compound	R	IC_50_ (μM)			
		K562 ^b^	KU812 ^c^	HT-29 ^d^	HCT116 ^e^
**T1**	4-CH_3_	22.05 ± 0.12	9.55 ± 0.17	7.10 ± 0.45	4.85 ± 0.32
**T2**	4-OCH_3_	>30	13.31 ± 0.39	8.78 ± 0.12	3.97 ± 0.53
**T3**	4-OC_2_H_5_	15.50 ± 1.09	17.19 ± 0.67	5.16 ± 0.11	2.47 ± 0.56
**T4**	4-CF_3_	8.60 ± 0.61	8.09 ± 0.91	2.87 ± 0.32	1.71 ± 0.46
**T5**	4-OCF_3_	>30	12.45 ± 0.02	3.64 ± 0.21	3.94 ± 0.33
**T6**	4-F	>30	16.90 ± 1.58	>30	4.39 ± 0.71
**T7**	4-Cl	>30	17.52 ± 1.77	>30	2.08 ± 0.54
**T8**	4-*t*-Bu	19.48 ± 0.12	12.02 ± 1.28	4.94 ± 0.25	6.51 ± 0.19
**T9**	2-CH_3_	>30	>30	>30	10.02 ± 0.93
**T10**	3-CH_3_	>30	>30	>30	7.62 ± 1.23
**T11**	2-OC_2_H_5_	>30	>30	>30	>30
**T12**	2-F	>30	>30	12.79 ± 0.98	6.14 ± 1.45
**T13**	2-Cl	>30	>30	13.88 ± 0.67	10.20 ± 0.87
**T14**	3-Cl	>30	>30	13.19 ± 0.53	9.83 ± 1.98
**T15**	2,4-*di*-CH_3_	>30	>30	>30	>30
**T16**	2,4-*di*-F	>30	>30	>30	>30
**T17**	2,6-*di*-F	>30	>30	>30	>30
**T18**	2,6-*di*-Cl	>30	>30	>30	>30
**Echinatin**	/	>30	13.77 ± 0.33	10.39 ± 0.87	4.54 ± 0.76
**Adriamycin**	/	4.22 ± 0.44	4.14 ± 0.53	2.70 ± 0.71	1.50 ± 0.19

^a^ IC_50_ value means ± SD, n = 3; ^b^ K562 (TCHu191, 37 ℃, 5 % CO_2_, IMDM + 10 % FBS + 1 % P/S); ^c^ KU812 (TCHu189, 37 ℃, 5 % CO_2_, 1640 + 10 % FBS + 1 % P/S); ^d^ HT-29 (SCSP-5032, 37 ℃, 5 % CO_2_, Mccoy’s 5A + 10 % FBS + 1 % P/S); ^e^ HCT116 (SCSP-5076, 37 ℃, 5 % CO_2_, 1640 + 10 % FBS + 1 % P/S).

**Table 2:** IC_50_^a^ values (μM) of Compound **T4** against HCT116 and CCC-HIE-2.

Compound	HCT116	CCC-HIE-2	SI
**T4**	1.71 ± 0.46	27.56 ± 1.37	16.1

**Table 3:** IC_50_ values for c-KIT inhibition by Compounds **T3**, **T4**, and **T8**.

Compound	IC_50_ (μM)
PLX647	0.068
**T3**	0.237
**T4**	0.178
**T8**	0.613

## Supplementary Materials

The ^1^H-NMR and ^13^C-NMR of Compounds **T1**–**T18** are available online. The supporting information can be downloaded at: https://www.mdpi.com/article/10.3390/ijms25042254/s1.
