# Supplementary material for: Synthesis and Antitumor Activity Evaluation of Novel Echinatin Derivatives with a 1,3,4-Oxadiazole Moiety
Source: Int J Mol Sci. 2024 Feb 13;25(4):2254. doi: 10.3390/ijms25042254 (PMC10889159; doi:10.3390/ijms25042254)
Supplement: Supplementary file 1 [file ijms-25-02254-s001.zip › ijms-2842239-supplementary.pdf]

Supporting Information

Reverse docking

The protein structure data utilized in this study were sourced from the PDBbind database, comprising a total of 17654 entries. Reverse docking was executed using Glide Dock, a specialized docking program developed by Schrödinger. The resulting analysis identified the top 50 structures, ranked based on their highest docking scores, as outlined below:

Table S1 Top 50 docking score of reverse docking.

Num.	PDB	Docking score	Num.	PDB	Docking score
1	5f1j	-16.97	26	4ckr	-13.54
2	5mym	-15.97	27	1fm9	-13.52
3	5ioy	-15.82	28	5vqx	-13.52
4	4csj	-15.35	29	5vqt	-13.50
5	3hfj	-14.61	30	3are	-13.49
6	4hvs	-14.60	31	2hz0	-13.48
7	3mlb	-14.49	32	5m7m	-13.43
8	5ipa	-14.41	33	2og8	-13.42
9	4m3e	-13.86	34	3ctq	-13.41
10	3n7h	-13.80	35	4m3f	-13.41
11	4ymj	-13.79	36	5q15	-13.39
12	3k22	-13.79	37	4m3d	-13.34
13	5ezh	-13.79	38	5vqq	-13.32
14	3g08	-13.76	39	4dlj	-13.29
15	4pms	-13.71	40	5f04	-13.27
16	6c0n	-13.71	41	4m3b	-13.24
17	3k84	-13.67	42	3fun	-13.19
18	6fni	-13.66	43	2vj8	-13.18
19	5f0h	-13.66	44	4at4	-13.18
20	2ofv	-13.64	45	4omk	-13.16
21	3qri	-13.62	46	5yqo	-13.15
22	4ono	-13.59	47	5vqr	-13.14
23	4mxc	-13.57	48	3ftz	-13.14
24	5mo8	-13.56	49	4i5h	-13.13
25	4ckr	-13.54	50	3ugc	-13.13

1H, 13C NMR and HRMS spectra

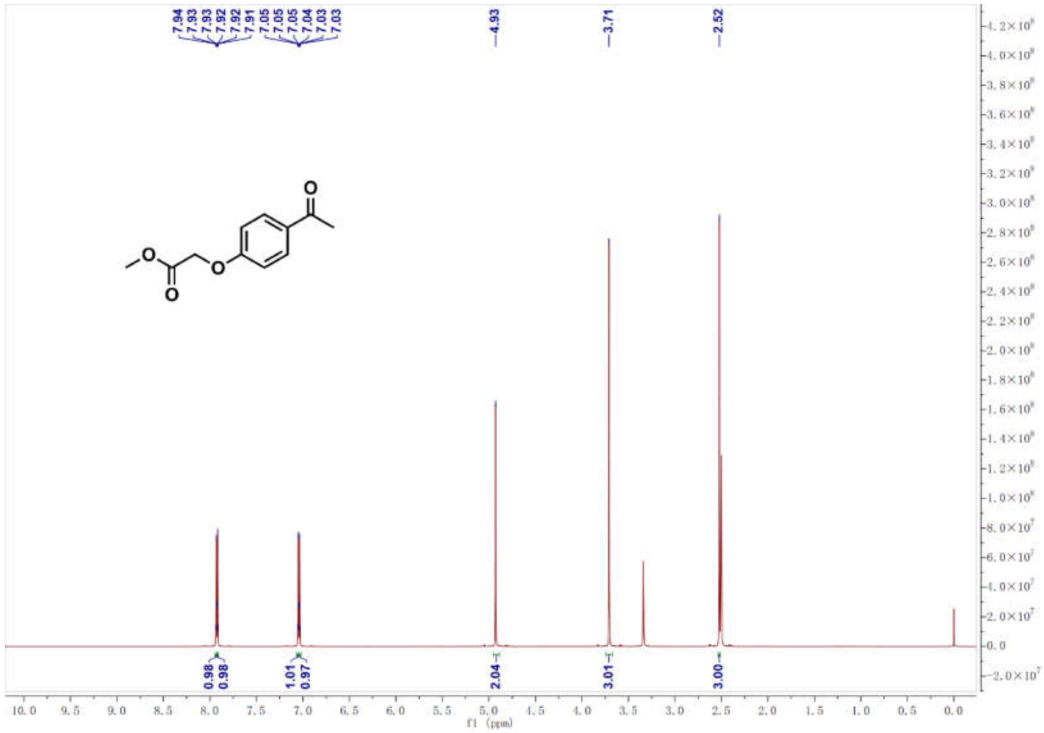

Figure S1. 1H NMR (600 MHz, DMSO-d_6) spectrum of compound 1

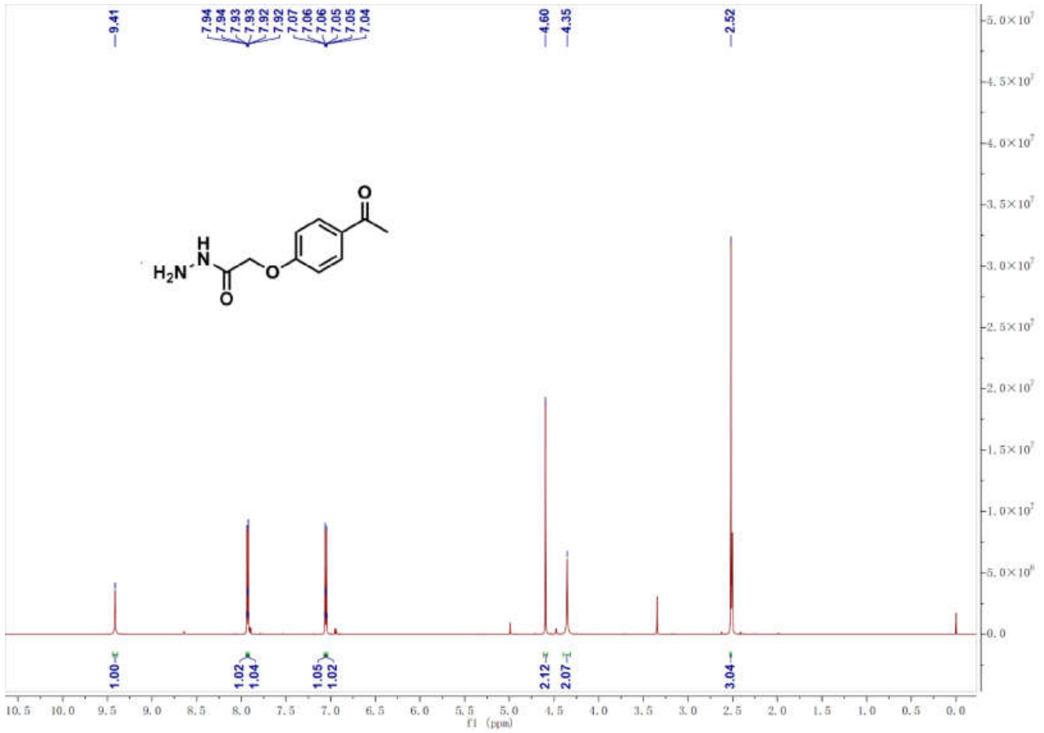

Figure S2. 1H NMR (600 MHz, DMSO-d_6) spectrum of compound 2

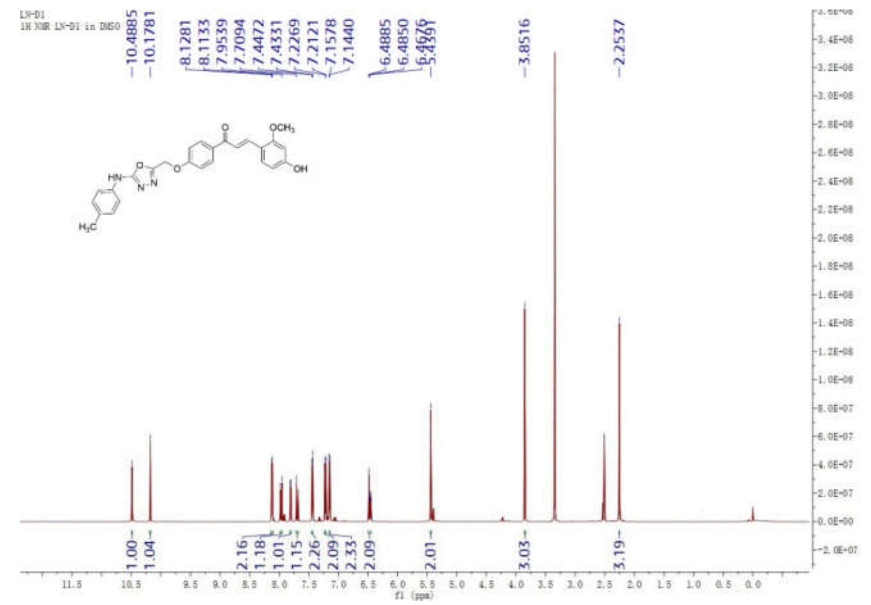

Figure S3. ^{1}H NMR (600 MHz, DMSO-d_6) spectrum of compound T1

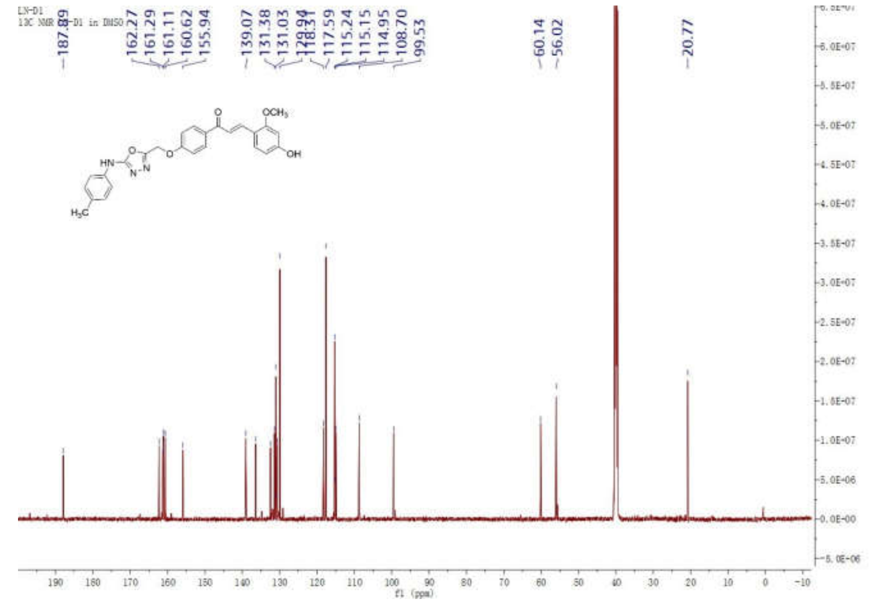

Figure S4. ^{13}C NMR (150 MHz, DMSO-d_6) spectrum of compound T1

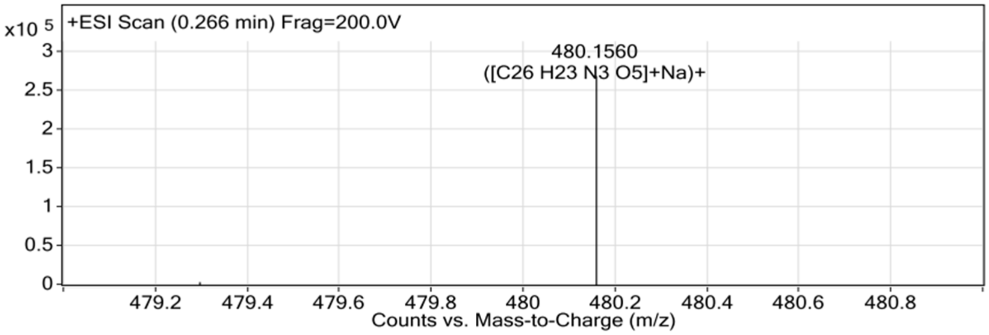

Figure S5. Mass spectrum of compound T1

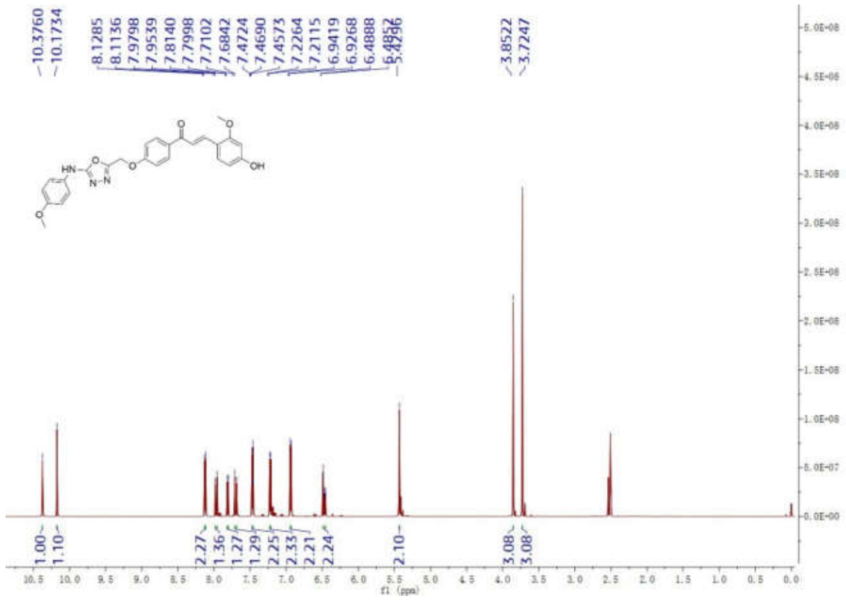

Figure S6. ^{1}H NMR (600 MHz, DMSO-d_6) spectrum of compound T2

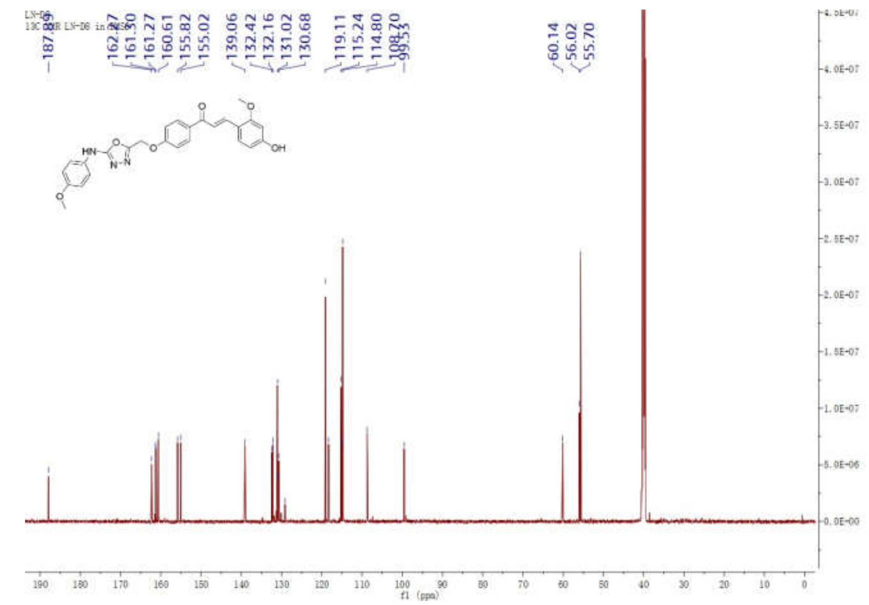

Figure S7. **^{13}C NMR (150 MHz, DMSO-*d*6) spectrum of compound T2**

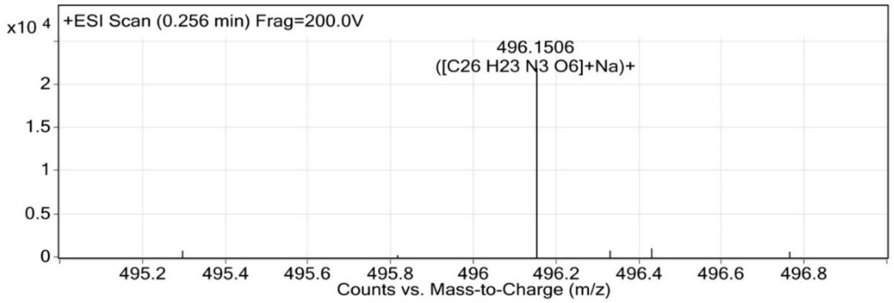

Figure S8. **Mass spectrum of compound T2**

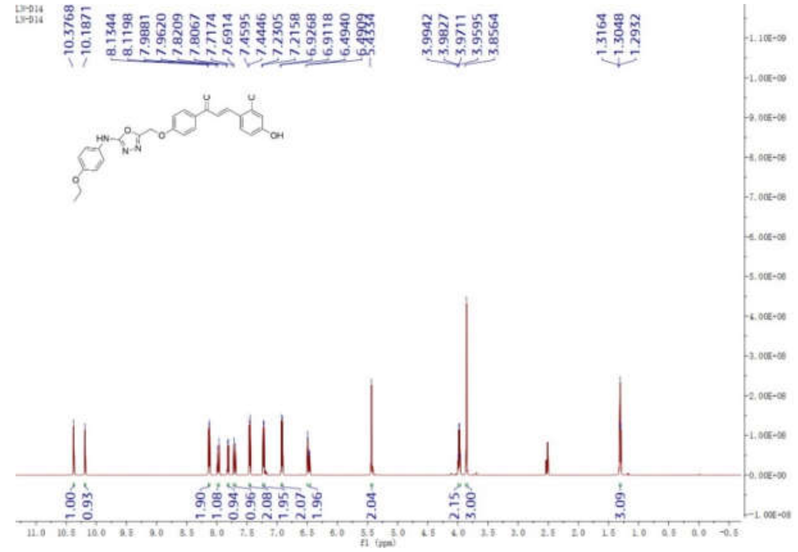

Figure S9. **^{1}H NMR (600 MHz, DMSO-*d*6) spectrum of compound T3**

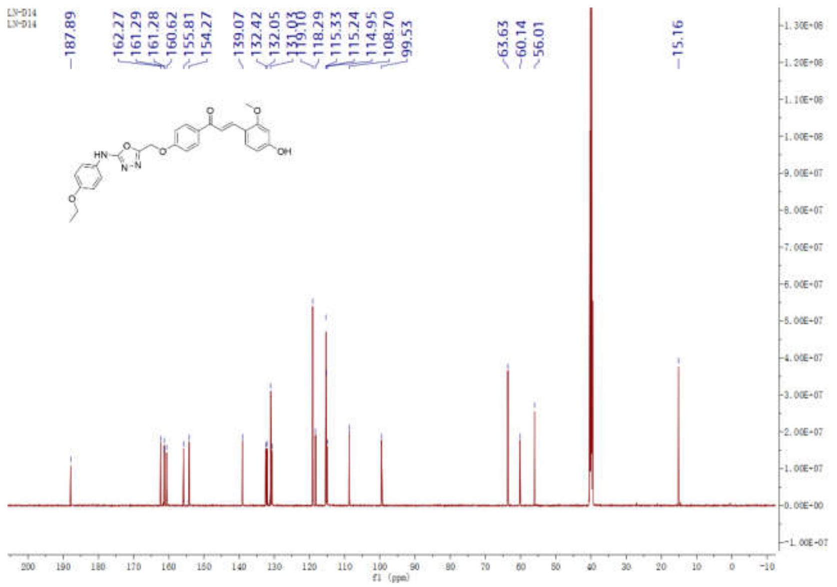

Figure S10. ^{13}C NMR (150 MHz, DMSO-d_6) spectrum of compound T3

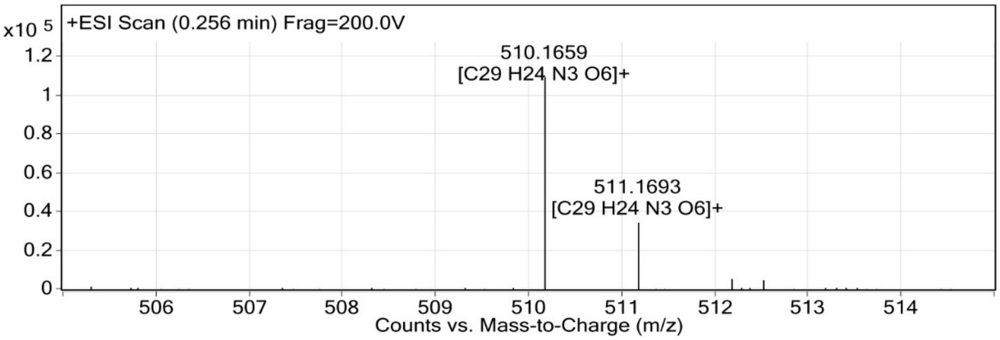

Figure S11. Mass spectrum of compound T3

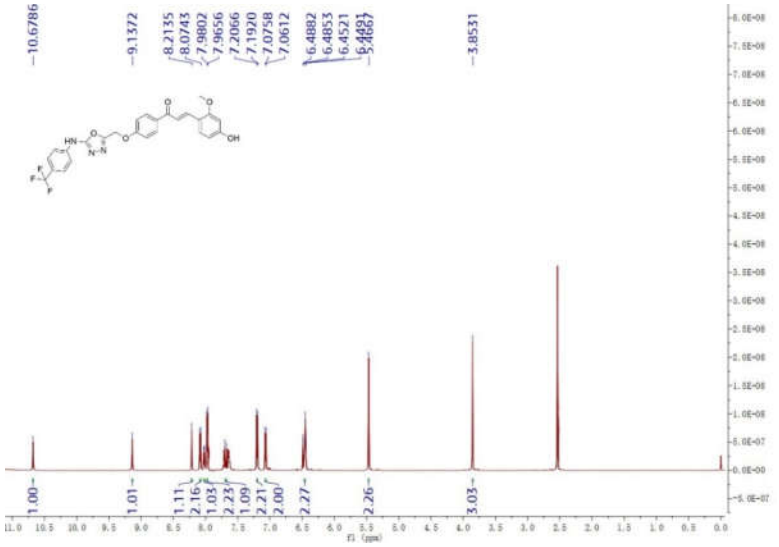

Figure S12. ^{1}H NMR (600 MHz, DMSO-d_6) spectrum of compound T4

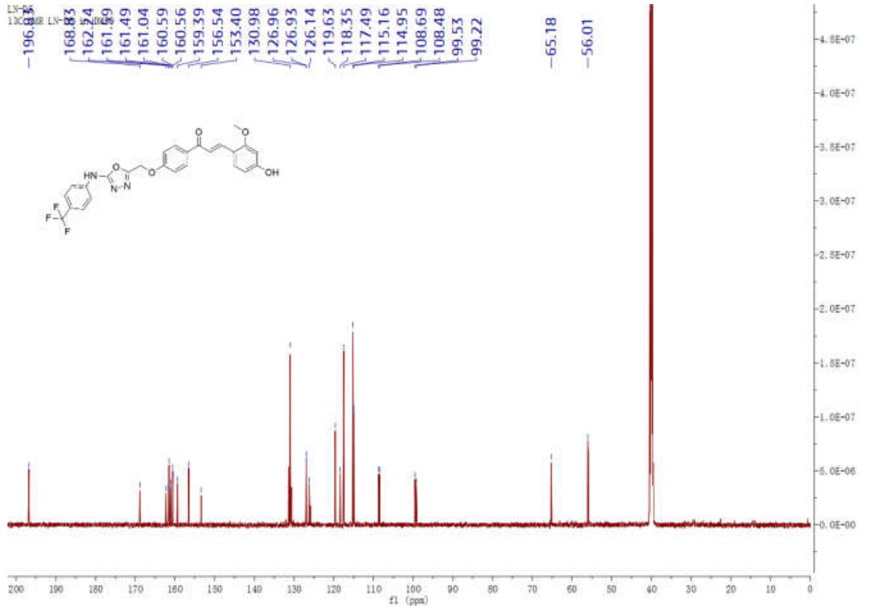

Figure S13. ^{13}C NMR (150 MHz, DMSO-*d*$_6$) spectrum of compound T4

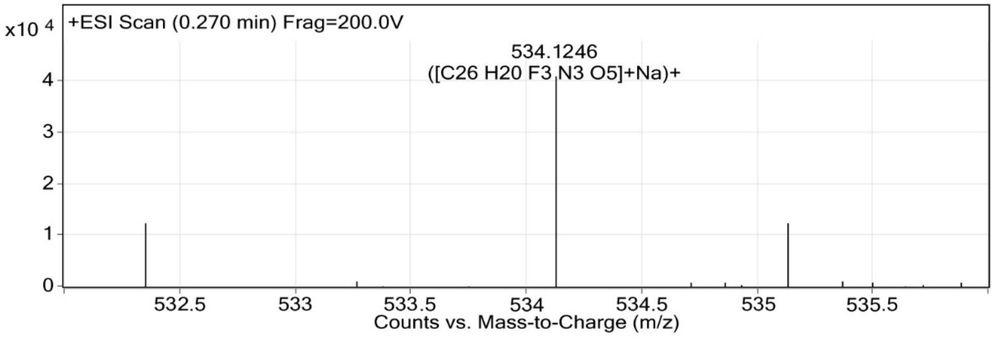

Figure S14. Mass spectrum of compound T4

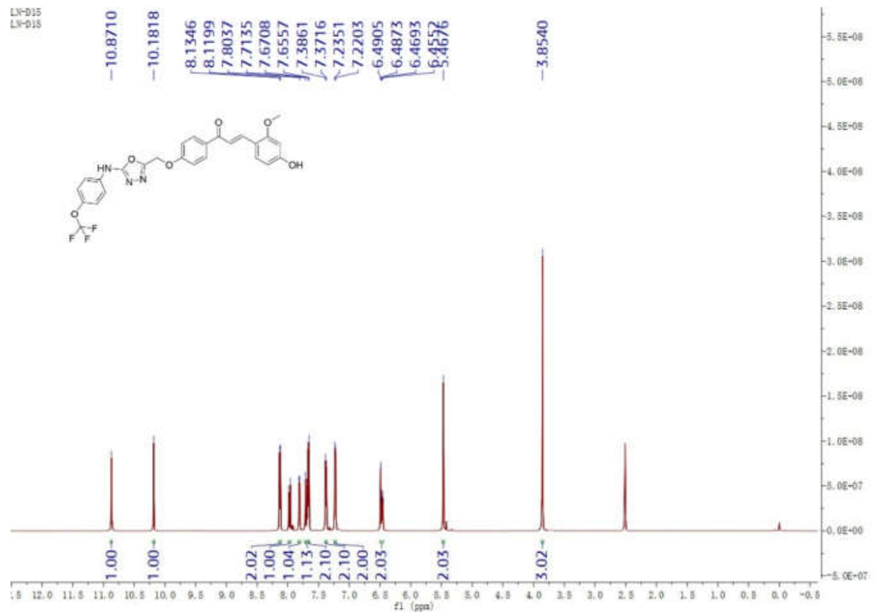

Figure S15. ^{1}H NMR (600 MHz, DMSO-d_6) spectrum of compound T5

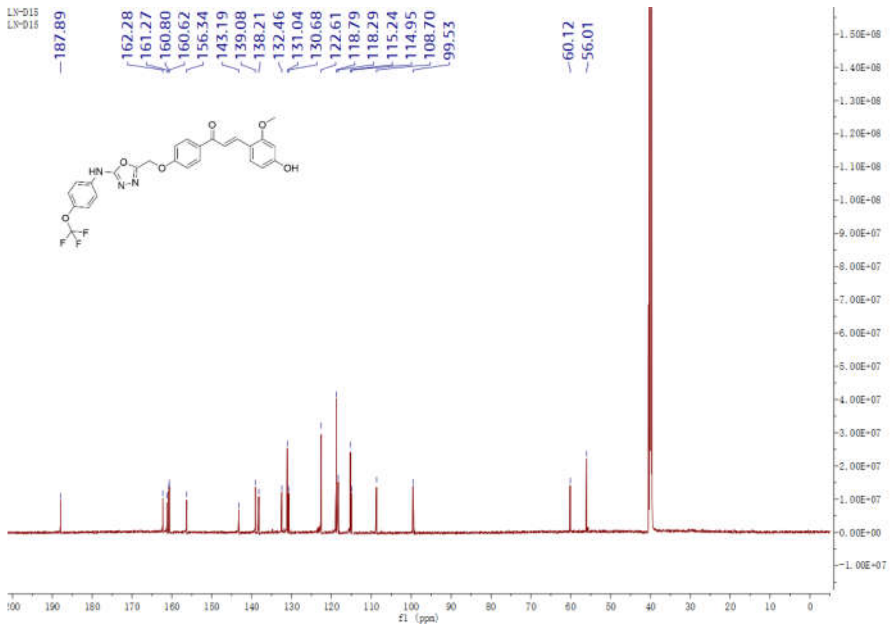

Figure S16. ^{13}C NMR (150 MHz, DMSO-d_6) spectrum of compound T5

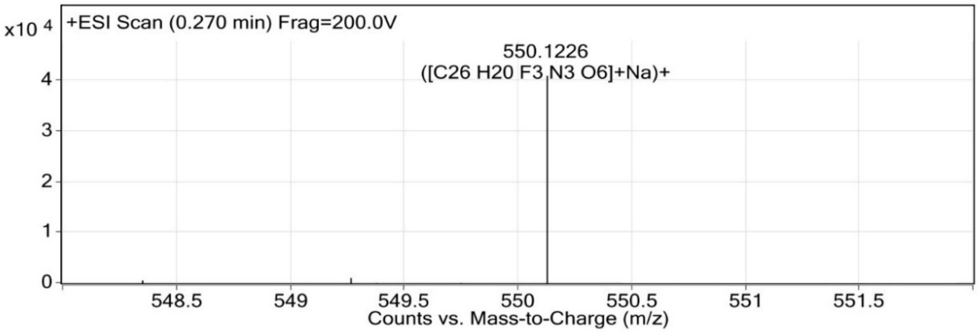

Figure S17. Mass spectrum of compound T5

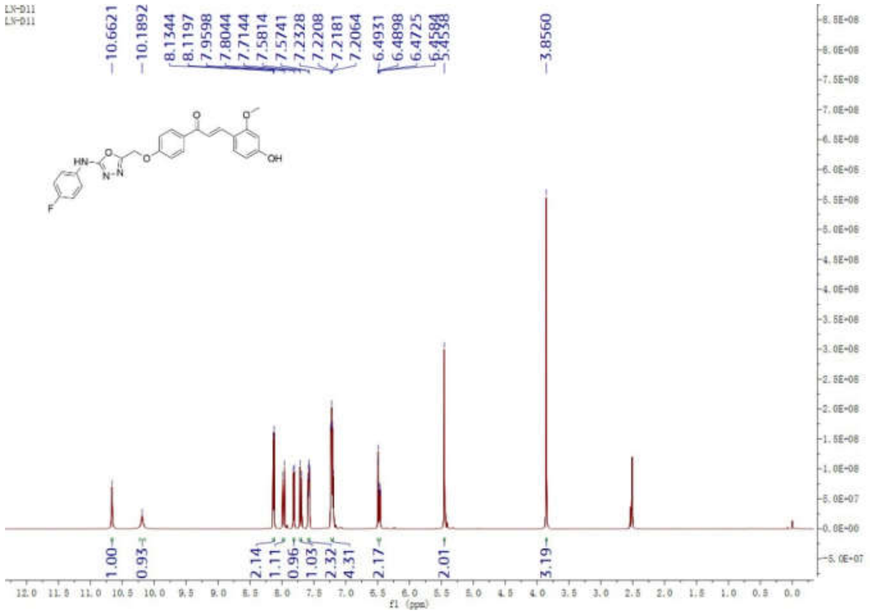

Figure S18. ^{1}H NMR (600 MHz, DMSO-d_6) spectrum of compound T6

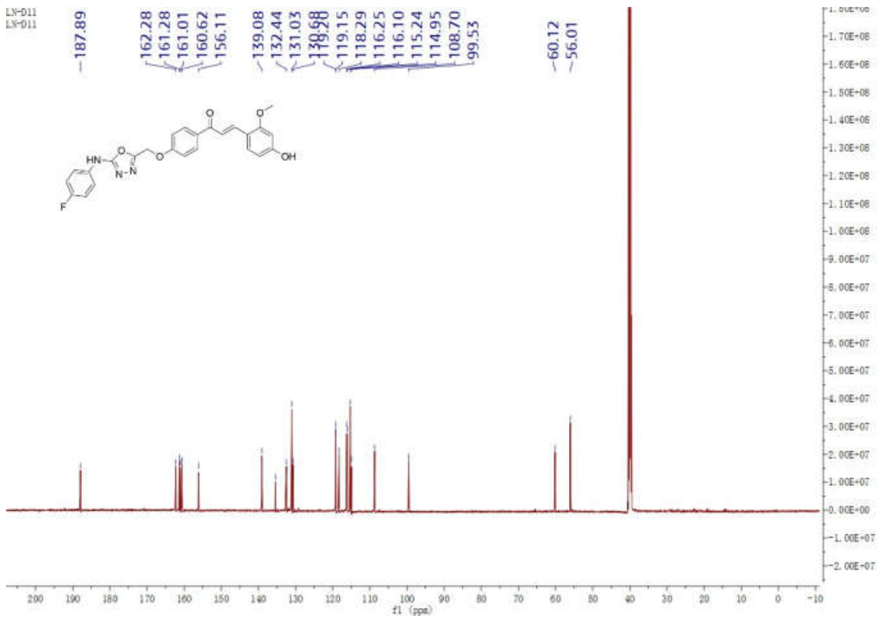

Figure S19. ^{13}C NMR (150 MHz, DMSO-d_6) spectrum of compound T6

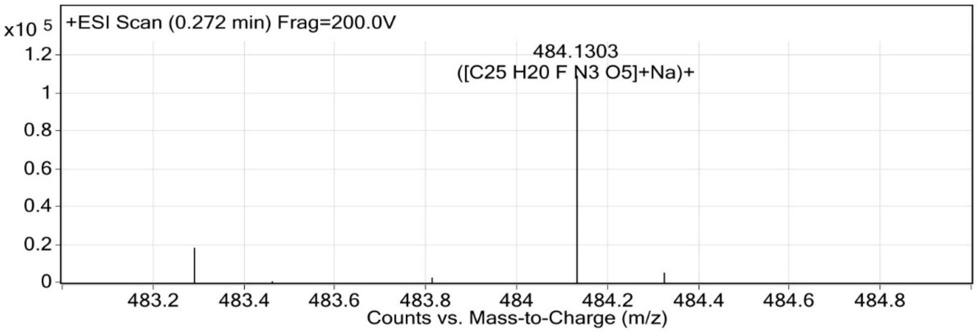

Figure S20. Mass spectrum of compound T6

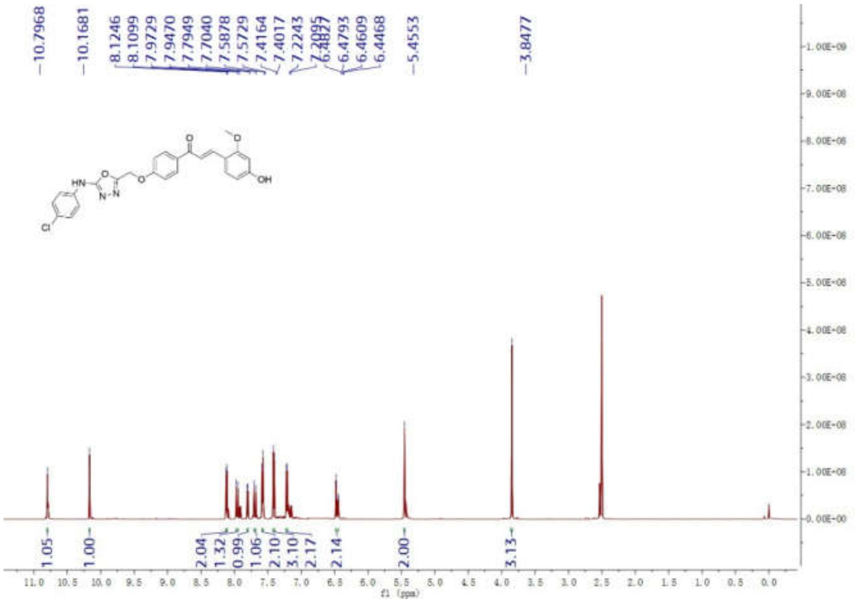

Figure S21. ^{1}H NMR (600 MHz, DMSO-d_6) spectrum of compound T7

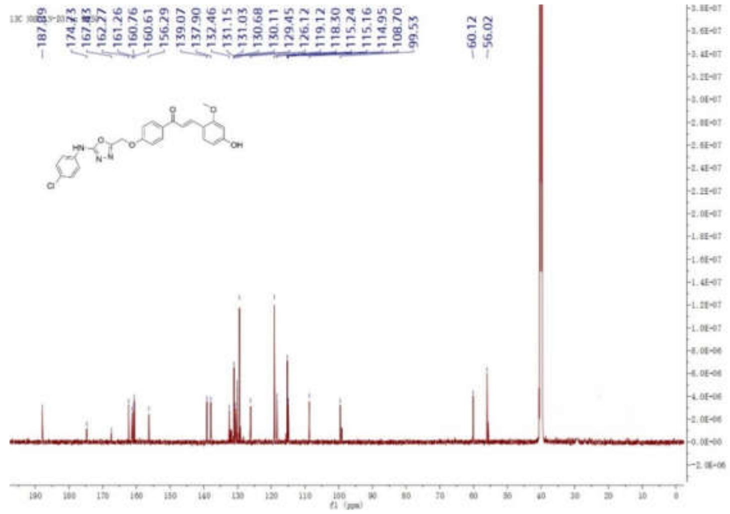

Figure S22. ^{13}C NMR (150 MHz, DMSO-d_6) spectrum of compound T7

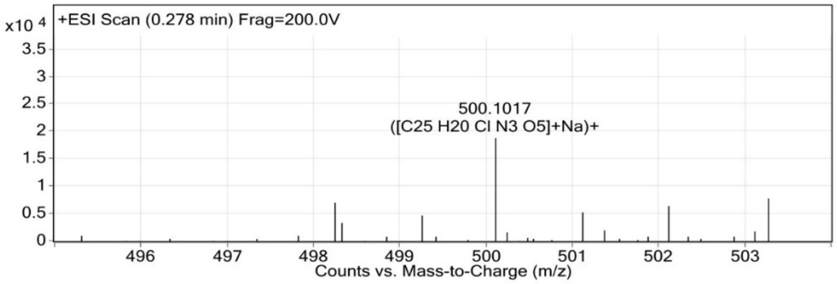

Figure S23. Mass spectrum of compound T7

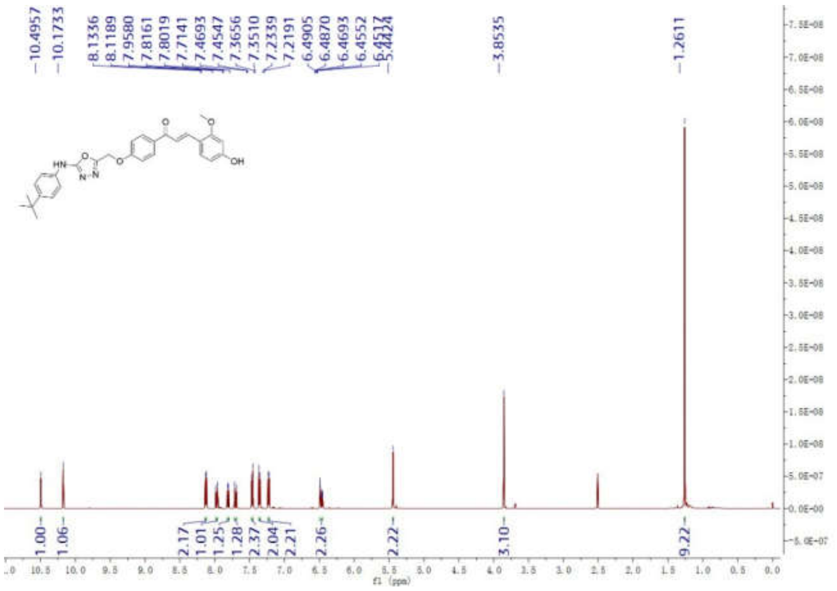

Figure S24. ^{1}H NMR (600 MHz, DMSO-d_6) spectrum of compound T8

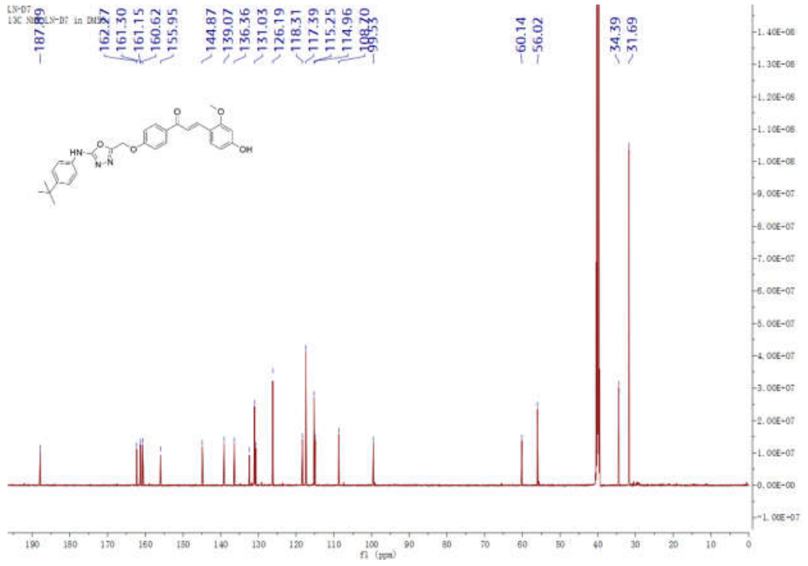

Figure S25. ^{13}C NMR (150 MHz, DMSO-d_6) spectrum of compound T8

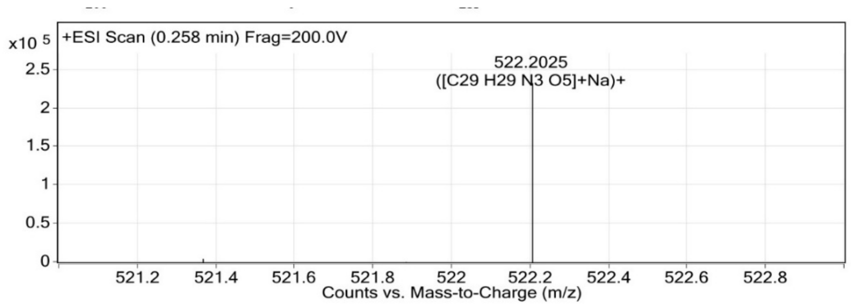

Figure S26. Mass spectrum of compound T8

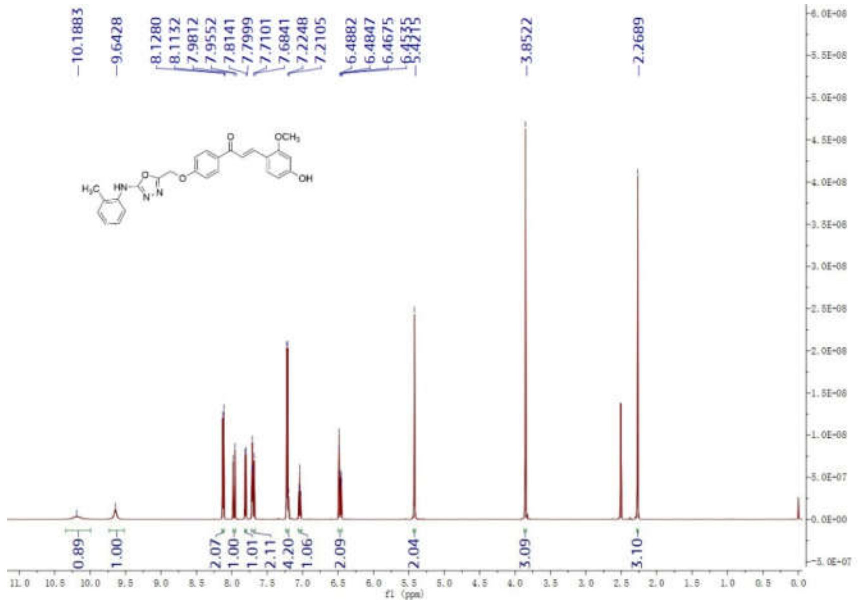

Figure S27. ^{1}H NMR (600 MHz, DMSO-d_6) spectrum of compound T9

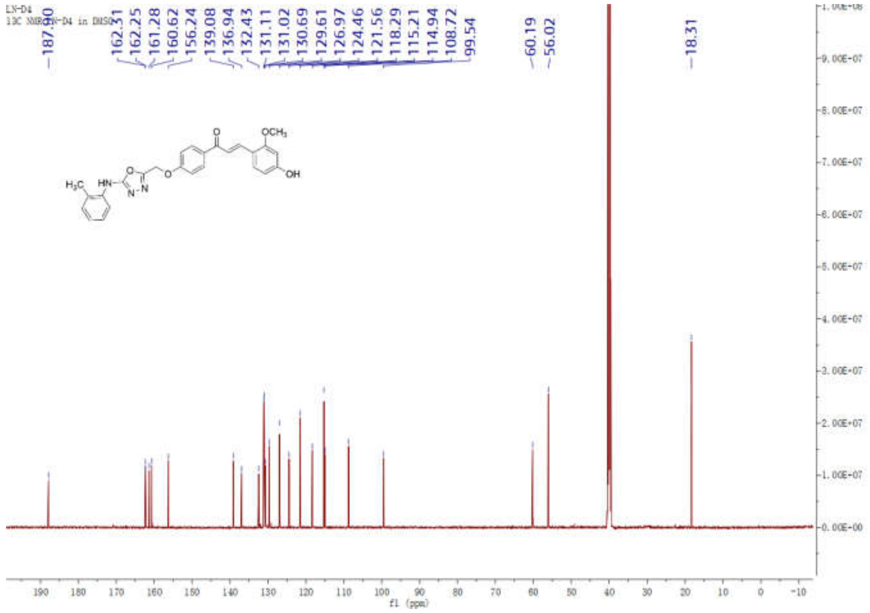

Figure S28. ^{13}C NMR (150 MHz, DMSO-d_6) spectrum of compound T9

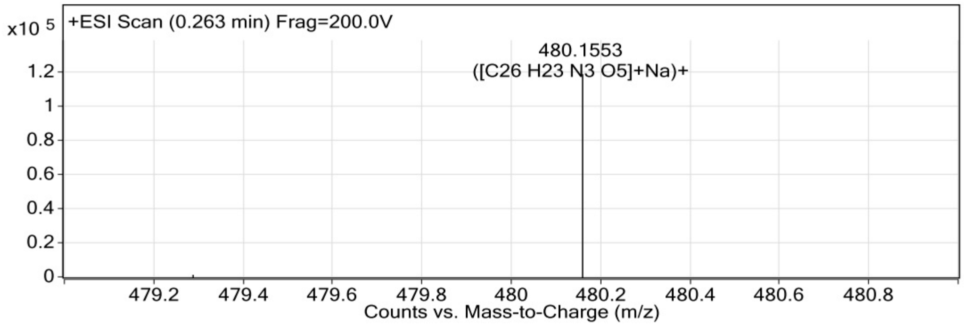

Figure S29. Mass spectrum of compound T9

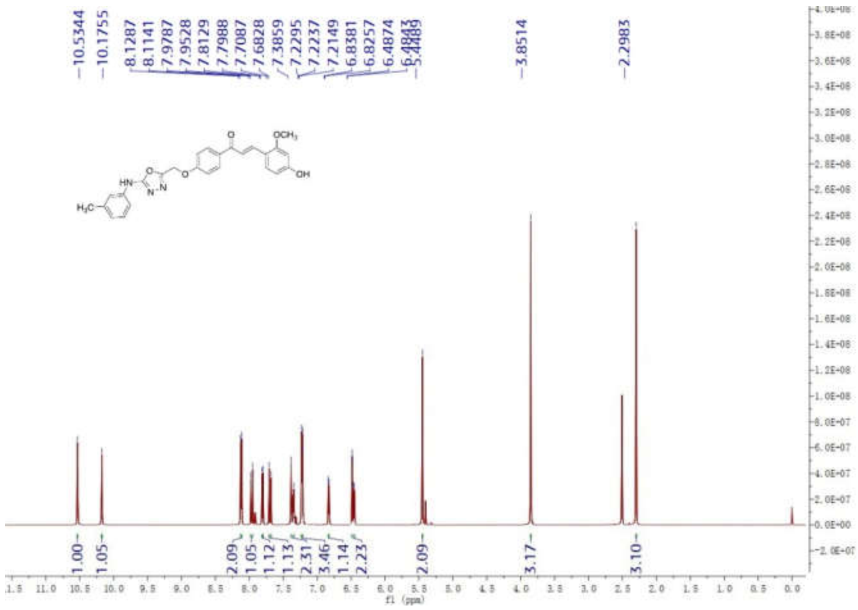

Figure S30. ^{1}H NMR (600 MHz, DMSO-d_6) spectrum of compound T10

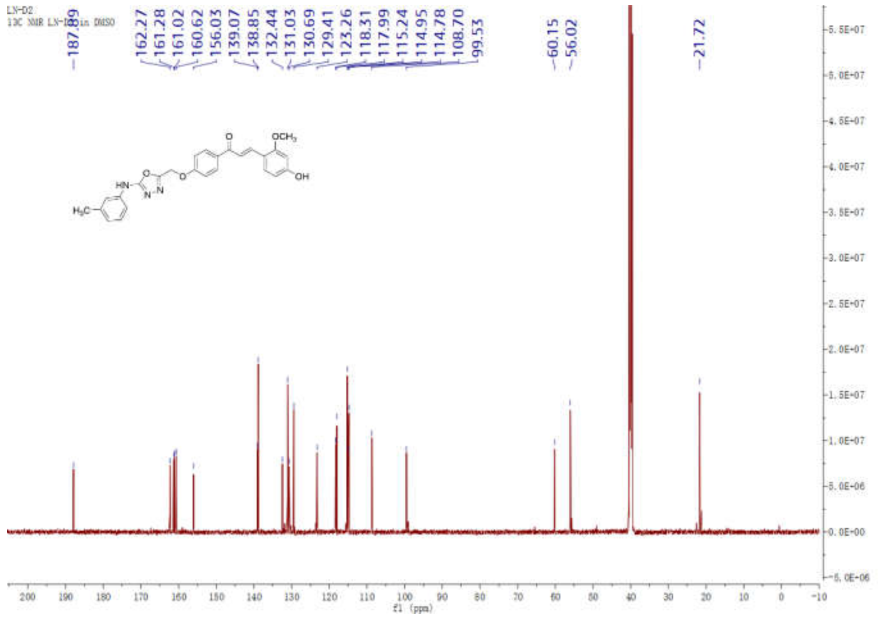

Figure S31. ^{13}C NMR (150 MHz, DMSO-d_6) spectrum of compound T10

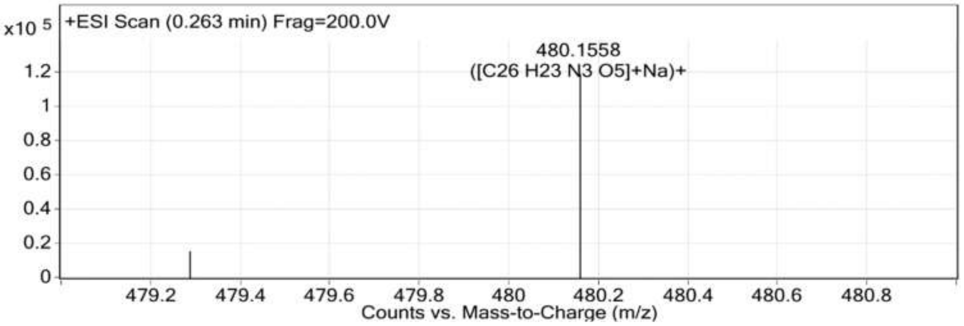

Figure S32. Mass spectrum of compound T10

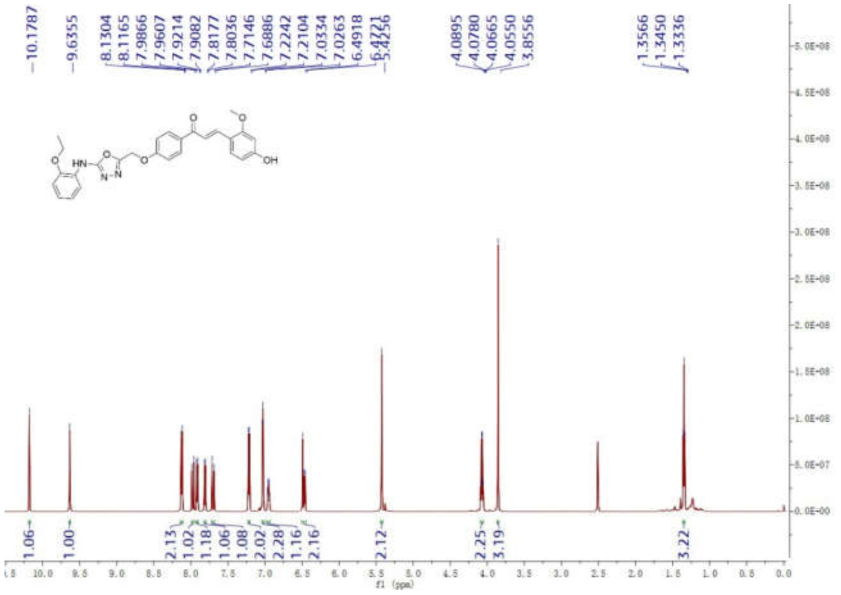

Figure S33. ^{1}H NMR (600 MHz, DMSO-d_6) spectrum of compound T11

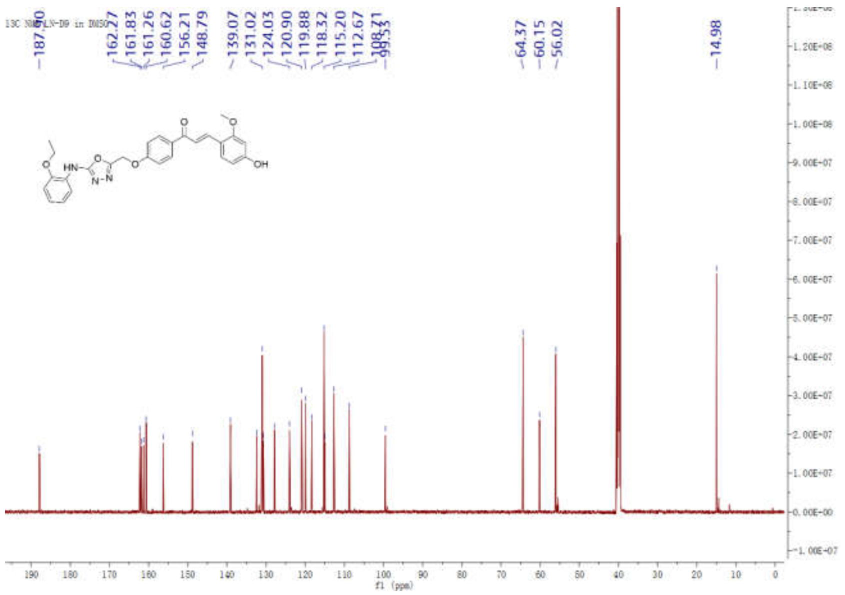

Figure S34. ^{13}C NMR (150 MHz, DMSO-d_6) spectrum of compound T11

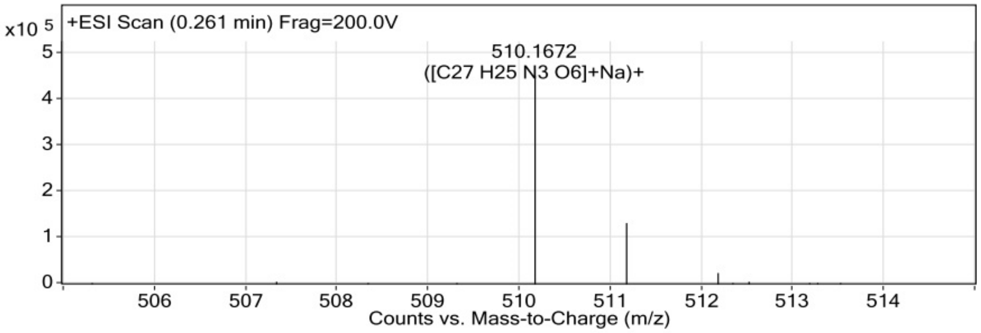

Figure S35. Mass spectrum of compound T11

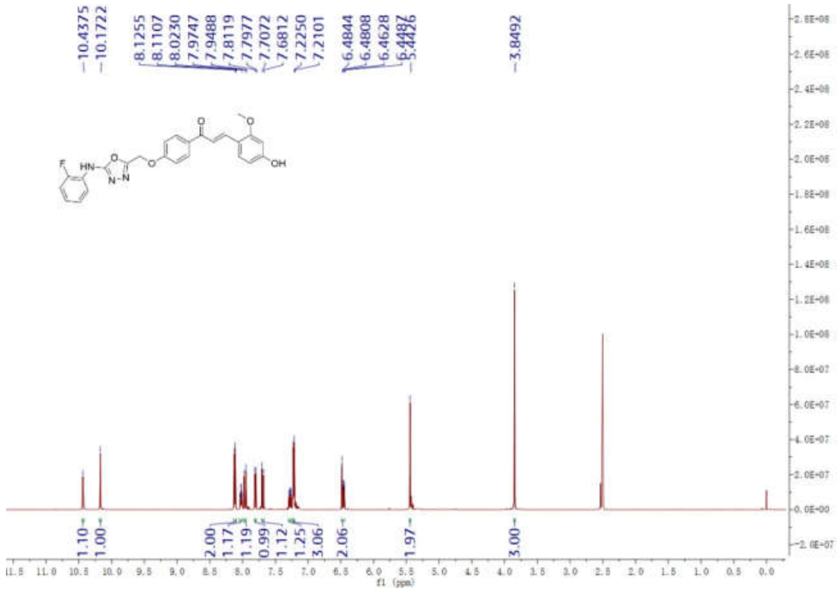

Figure S36. ^{1}H NMR (600 MHz, DMSO-d_6) spectrum of compound T12

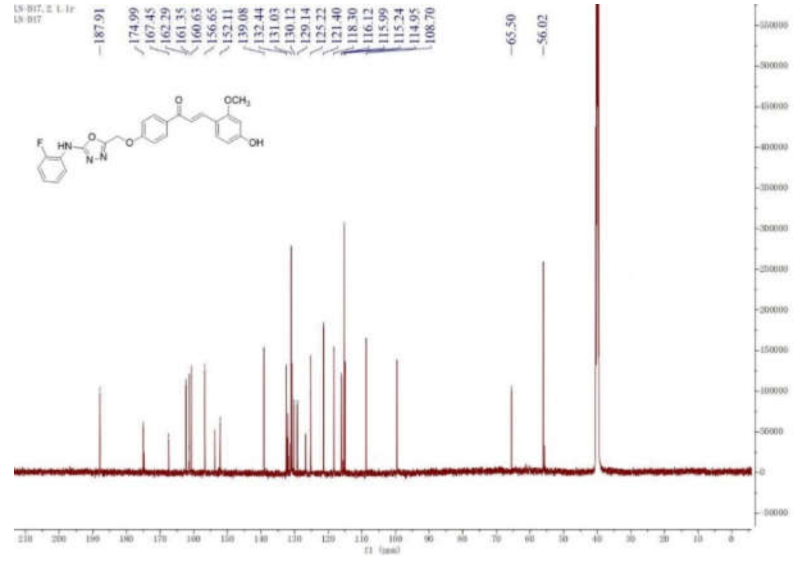

Figure S37. ^{13}C NMR (150 MHz, DMSO-d_6) spectrum of compound T12

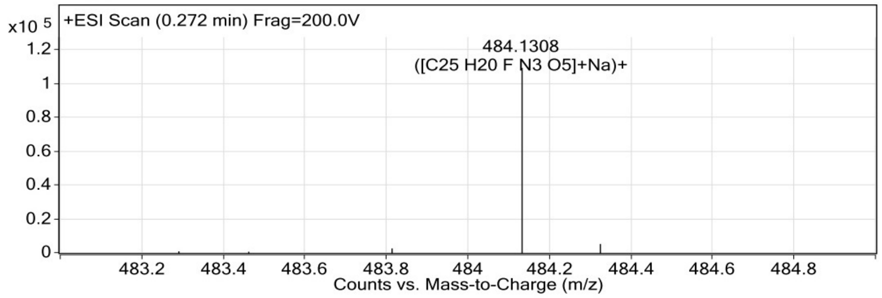

Figure S38. Mass spectrum of compound T12

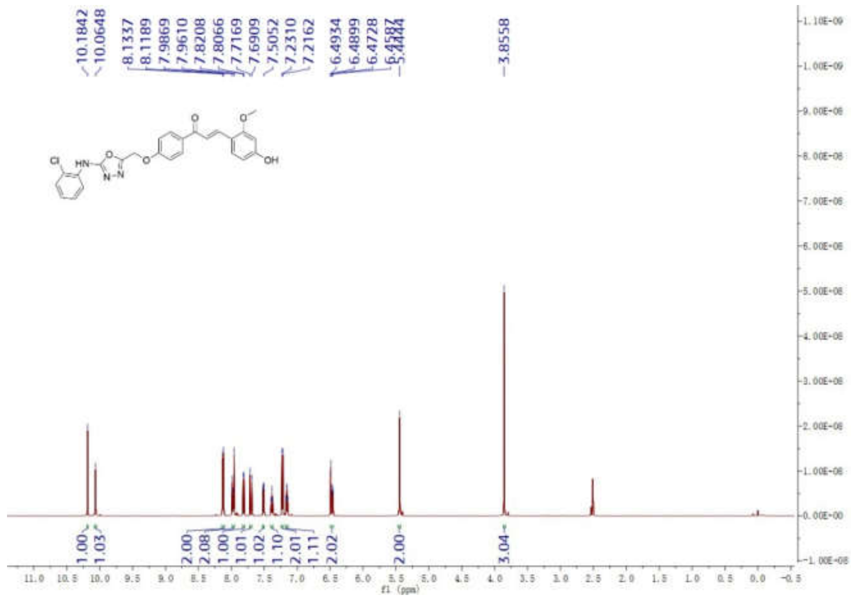

Figure S39. ^{1}H NMR (600 MHz, DMSO-d_6) spectrum of compound T13

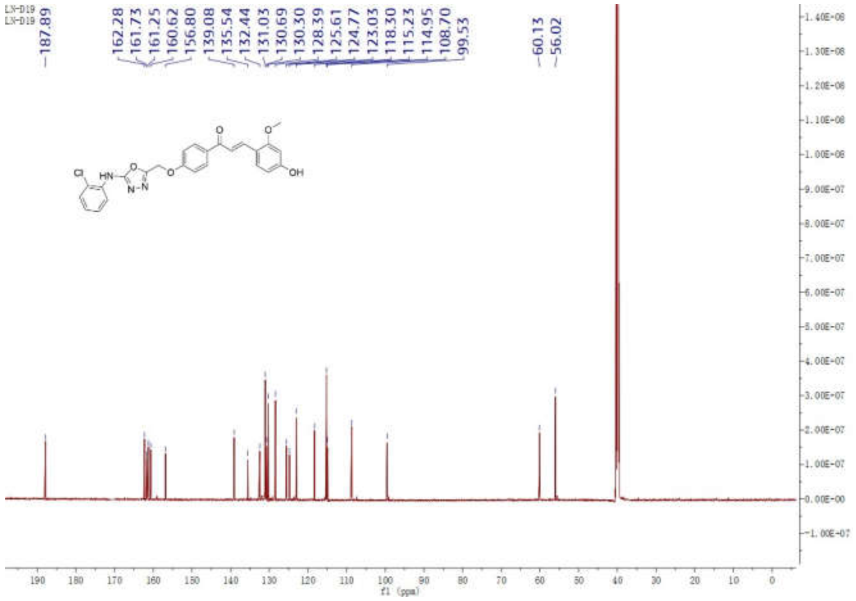

Figure S40. ^{13}C NMR (150 MHz, DMSO-d_6) spectrum of compound T13

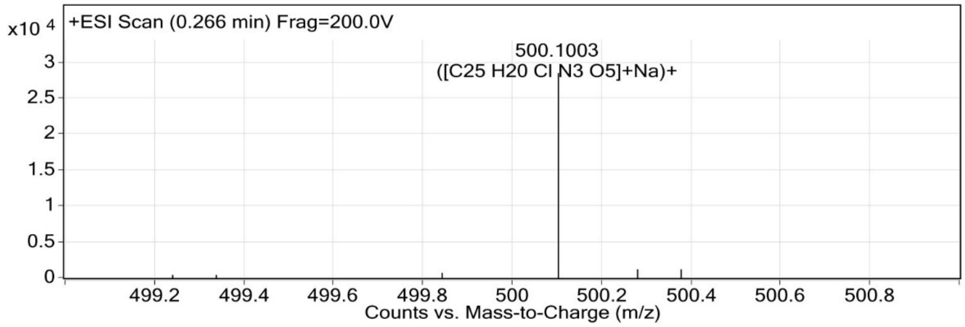

Figure S41. **Mass spectrum of compound T13**

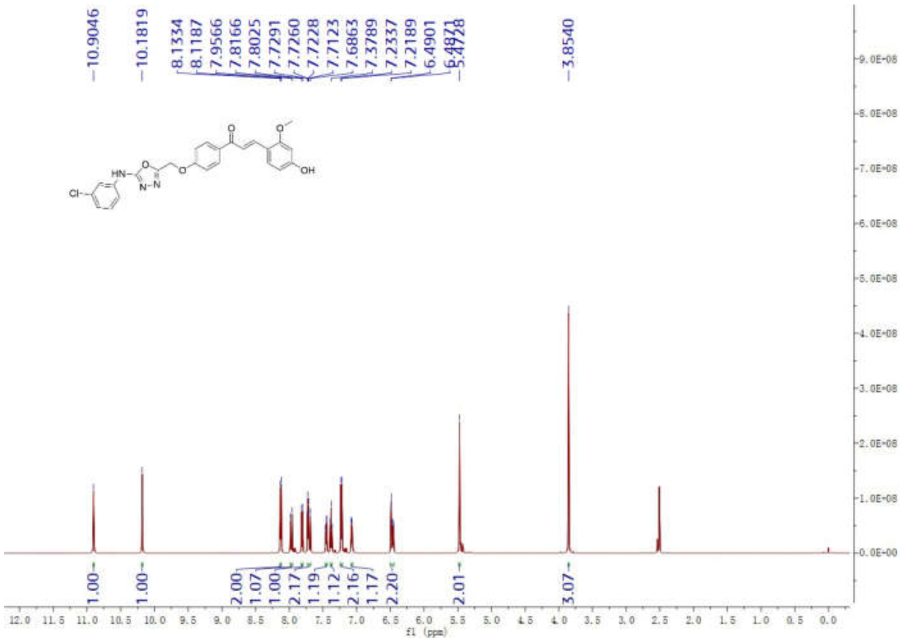

Figure S42. **^{1}H NMR (600 MHz, DMSO-d_6) spectrum of compound T14**

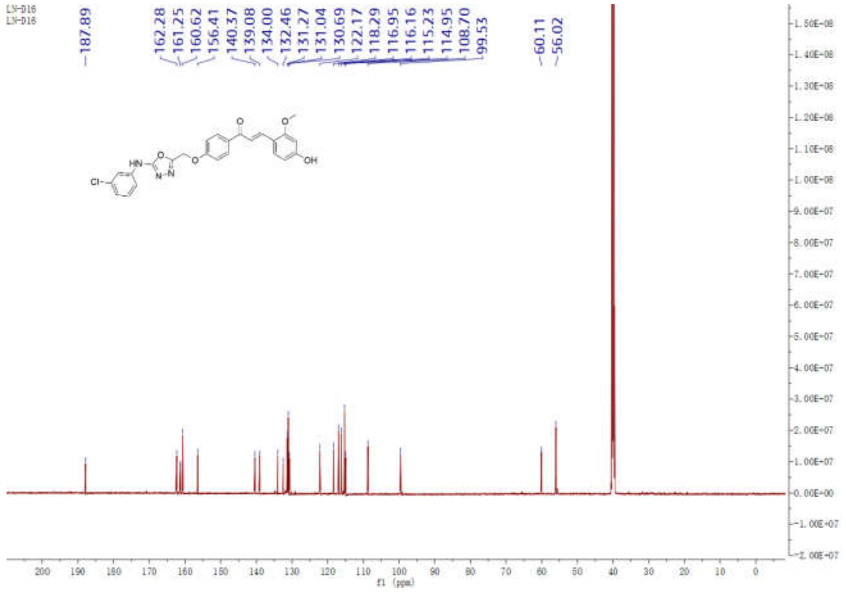

Figure S43. ^{13}C NMR (150 MHz, DMSO-d_6) spectrum of compound T14

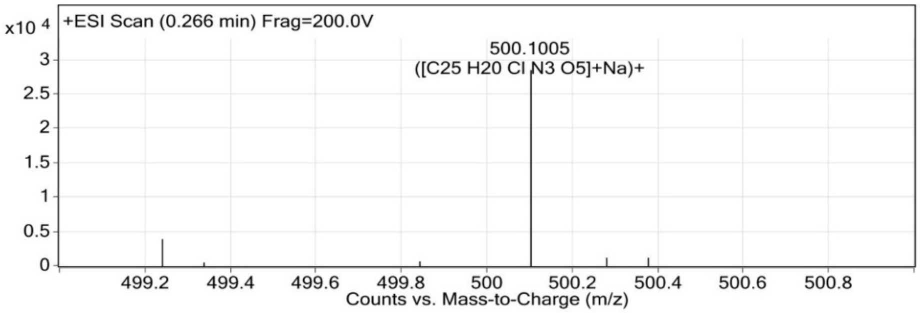

Figure S44. Mass spectrum of compound T14

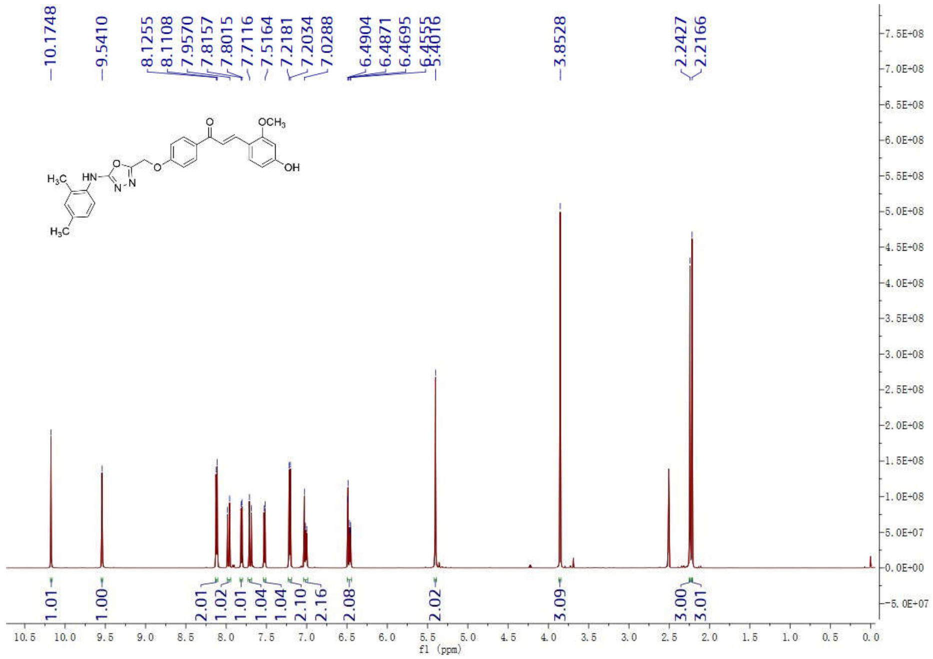

Figure S45. ^{1}H NMR (600 MHz, DMSO-d_6) spectrum of compound T15

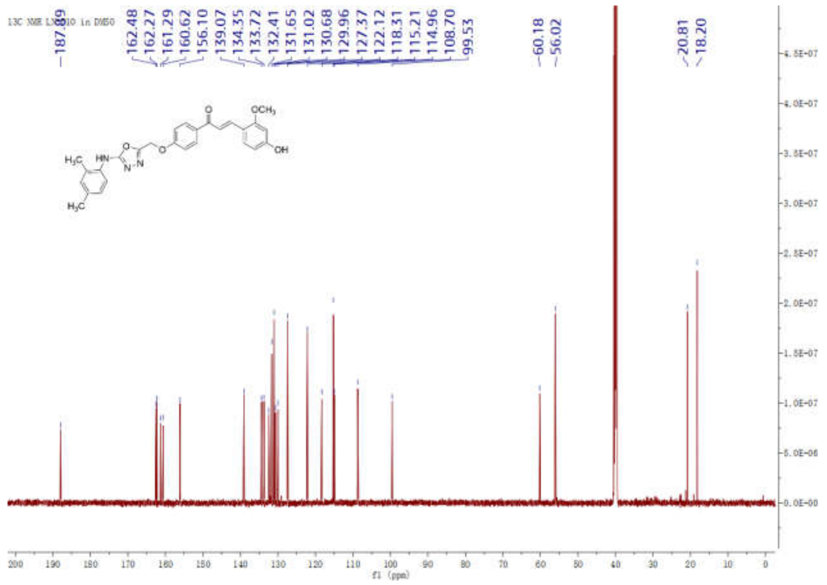

Figure S46. ^{13}C NMR (150 MHz, DMSO-d_6) spectrum of compound T15

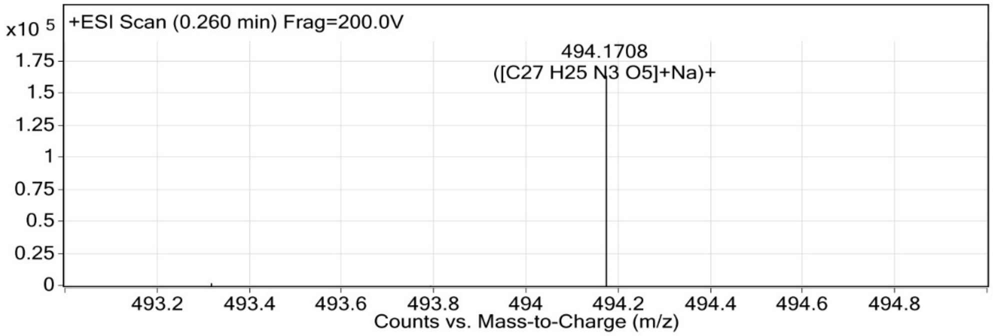

Figure S47. **Mass spectrum of compound T15**

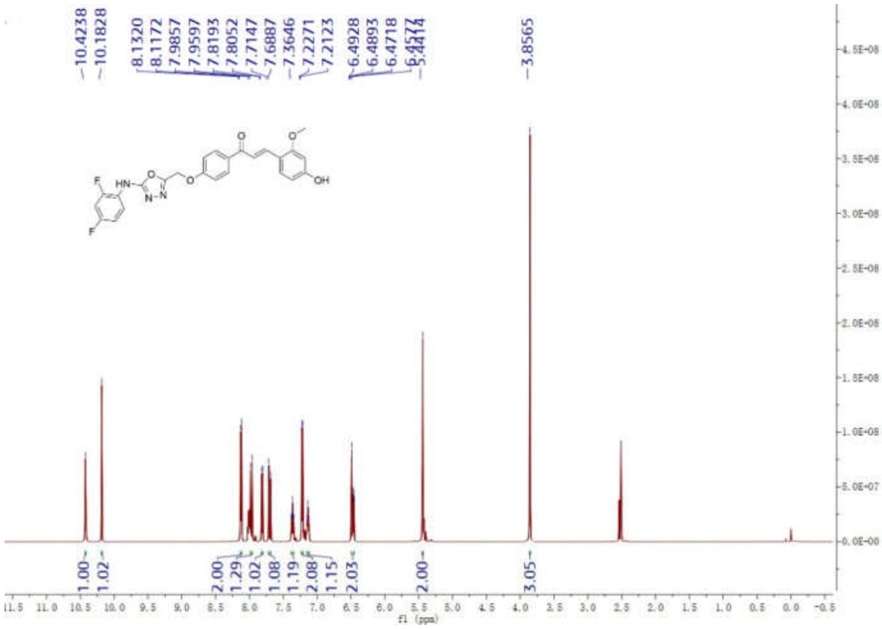

Figure S48. **^{1}H NMR (600 MHz, DMSO-d_6) spectrum of compound T16**

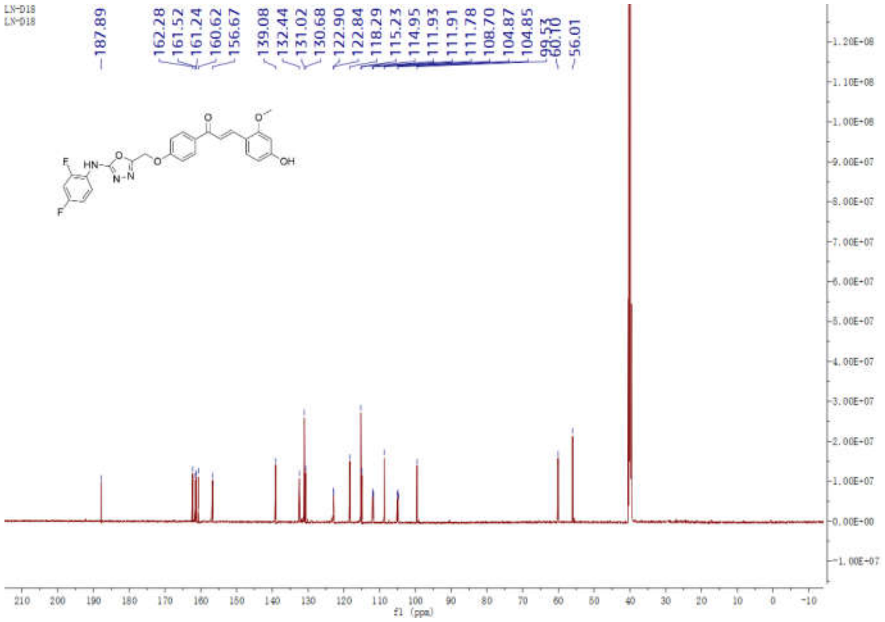

Figure S49. ^{13}C NMR (150 MHz, DMSO-d_6) spectrum of compound T16

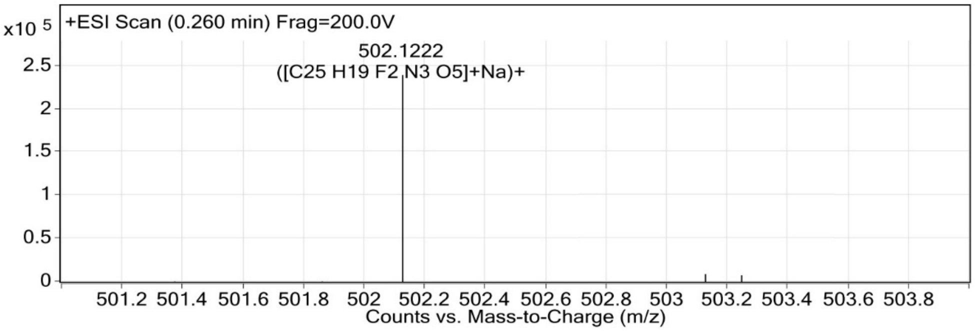

Figure S50. Mass spectrum of compound T16

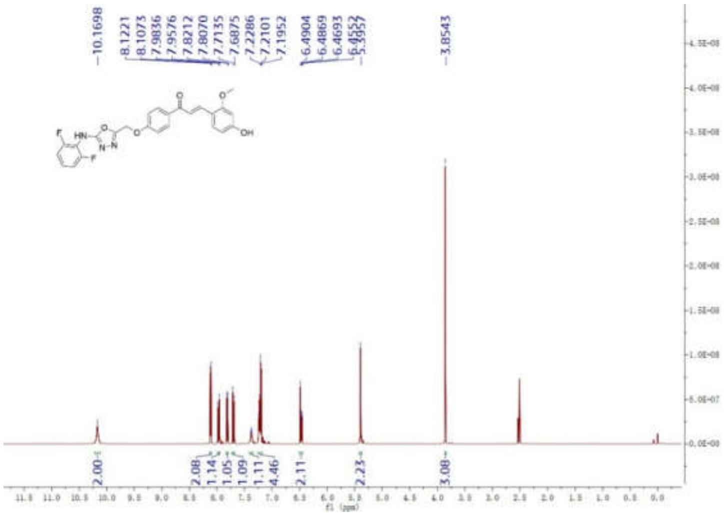

Figure S51. ^{1}H NMR (600 MHz, DMSO-d_6) spectrum of compound T17

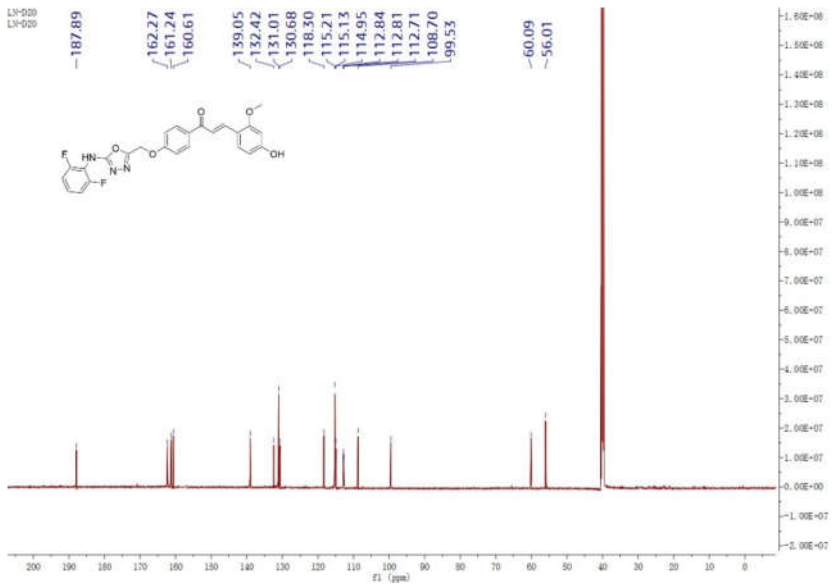

Figure S52. ^{13}C NMR (150 MHz, DMSO-d_6) spectrum of compound T17

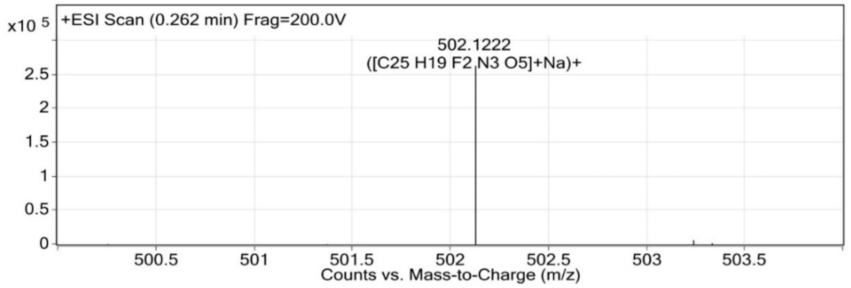

Figure S53. Mass spectrum of compound T17

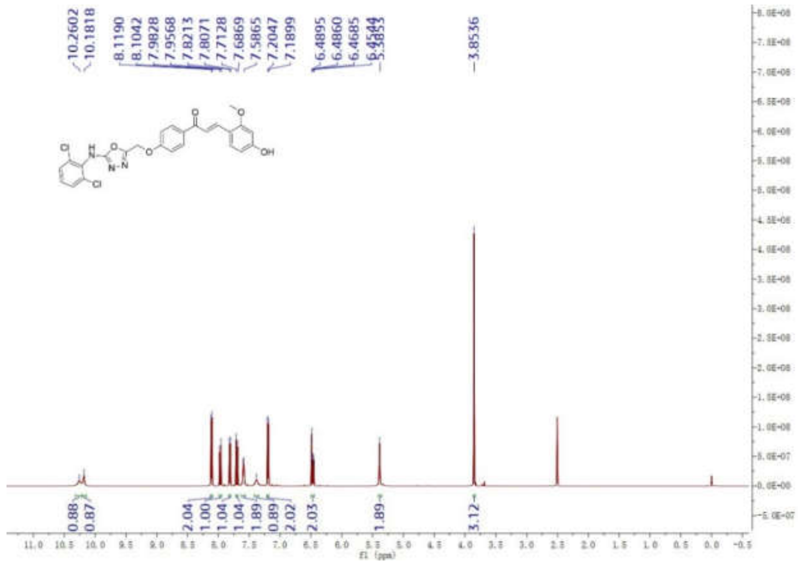

Figure S54. ^{1}H NMR (600 MHz, DMSO-d_6) spectrum of compound T18

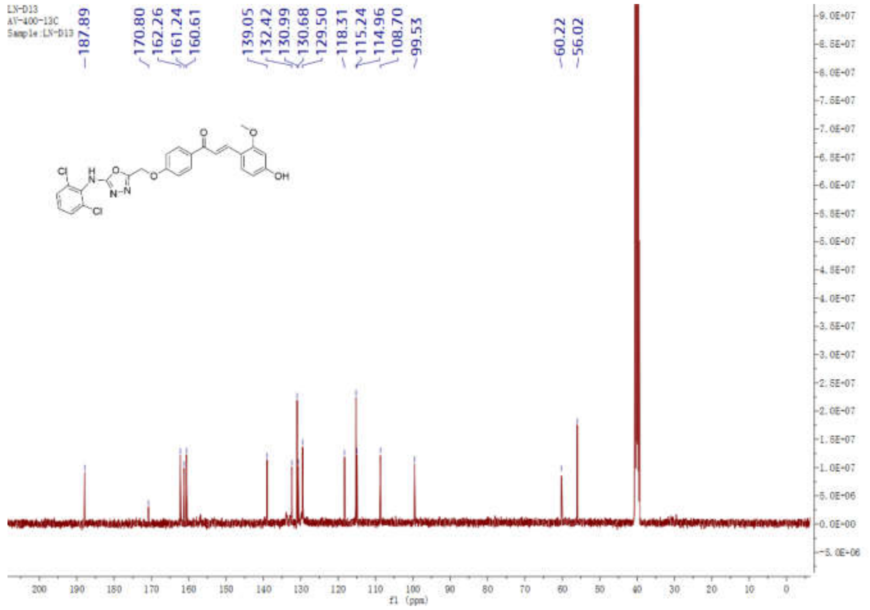

Figure S55. ^{13}C NMR (150 MHz, DMSO-d_6) spectrum of compound 18

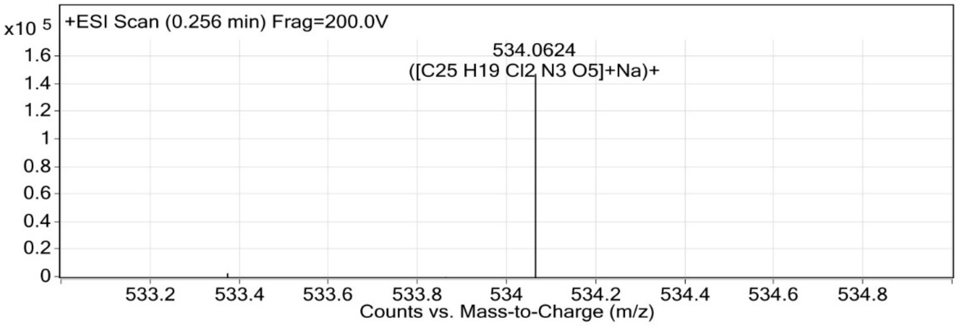

Figure S56. Mass spectrum of compound T18
